# Non-Thermal Plasma Couples Oxidative Stress to TRAIL Sensitization through DR5 Upregulation

**DOI:** 10.3390/ijms21155302

**Published:** 2020-07-26

**Authors:** Soon Young Hwang, Ngoc Hoan Nguyen, Tae Jung Kim, Youngsoo Lee, Mi Ae Kang, Jong-Soo Lee

**Affiliations:** 1 Department of Life Sciences, College of Natural Sciences, Ajou University, Suwon 16499, Korea; hwang630@ajou.ac.kr (S.Y.H.); hoanbiology@gmail.com (N.H.N.); 2 Department of Electrical and Computer Engineering, College of Information and Technology, Ajou University, Suwon 16499, Korea; prosecutor33@ajou.ac.kr; 3 Department of Biomedical Sciences, Ajou University Graduate School of Medicine, Suwon 16499, Korea; ysoolee@ajou.ac.kr

**Keywords:** plasma-activated medium, TRAIL, DR5, apoptosis, ROS/RNS

## Abstract

Tumor necrosis factor-related apoptosis-inducing ligand (TRAIL) induces apoptosis in various tumor cells without affecting most normal cells. Despite being in clinical testing, novel strategies to induce TRAIL-mediated apoptosis are in need to overcome cancer cell unresponsiveness and resistance. Plasma-activated medium (PAM) markedly stimulates reactive oxygen/nitrogen species (ROS/RNS)-dependent apoptosis in cancer cells. We investigate the capability of PAM and TRAIL (PAM/TRAIL) combination therapy to overcome TRAIL resistance and improve the anticancer efficacy of TRAIL. The combinatorial treatment of PAM and TRAIL shows synergistic effects on growth inhibition in TRAIL-resistant cancer cells via augmented apoptosis by two attributes. DR5 (TRAIL-R2) transcription by CHOP is upregulated in a PAM-generated ROS/RNS-dependent manner, and PAM itself upregulates PTEN expression mediated by suppression of miR-425 which is involved in Akt inactivation, leading to increased apoptosis induction. Treatment of cancer cell lines with the antioxidant N-acetylcysteine reduces the extent of membrane dysfunction and the expression of both CHOP-DR5 and miR-425-PTEN axes, attenuating PAM/TRAIL-induced cancer cell apoptosis. These data suggest that PAM/TRAIL treatment is a novel approach to sensitizing cancer cells to TRAIL-induced apoptosis and overcoming TRAIL resistance. PAM is a promising candidate for further investigations as a chemotherapeutic sensitizer in the treatment of cancer.

## 1. Introduction

Apoptosis can be triggered through both intrinsically and extrinsically initiated pathways. Most current chemotherapeutic strategies target the dysregulation of apoptotic pathways in cancer cells. Intrinsic pathways are initiated at the mitochondria level in a p53-dependent manner [1]. Conventional radio- and chemotherapies aim mainly at the p53-dependent intrinsic apoptotic pathway. However, more than half of human cancers carry the loss of p53 function, and are either initially resistant or eventually acquire resistance to these treatments. Thus many cancers continue to survive and thrive because of the lack of p53 function in intrinsic cellular apoptotic mechanisms. In contrast, p53 appears to be dispensable for extrinsic apoptotic pathways in most cancers. Extrinsic pathways are triggered by the binding of death ligands to death receptors (DRs) found in the cellular membrane. DRs contain death domains endowing these receptors a role in apoptosis, among other non-apoptotic roles. Hence, DR-mediated apoptosis may represent a better target for the treatment of cancers that harbor p53 mutations.

TNF-related apoptosis-inducing ligand (TRAIL, also known as Apo2 ligand) is a member of the tumor necrosis factor (TNF) family of cytokines that binds to DRs to induce apoptosis [1]. TRAIL could be used as a chemotherapeutic agent because it induces apoptosis in cancer cells but not in normal cells [2]. However, the promising preclinical results have not successfully translated into clinical trials [3,4,5], since most primary cancers and multiple cancer cell lines are TRAIL-resistant [5]. However, TRAIL can trigger non-apoptotic signaling pathways in certain TRAIL-resistant cancer cells [5]. TRAIL-sensitizing strategies targeting TRAIL-activated non-apoptotic pathways could be effective in overcoming TRAIL resistance in cancer cells.

TRAIL triggers extrinsic apoptosis by binding to the death receptors TRAIL-R1 (DR4) and TRAIL-R2 (DR5, also called Apo2, KILLER, or TRICK2). However, several factors account for cancer resistance to apoptotic and non-apoptotic TRAIL signalings, which may provide opportunities to overcome TRAIL resistance. TRAIL-related receptors TRAIL-R3, TRAIL-R4, and osteoprotegerin (TRAIL-R5 or OPG) lack death domains and serve as decoy receptors [6,7]. These decoy receptors compete with DR4 and DR5 for TRAIL-binding, and reducing opportunities for TRAIL induction of apoptotic pathways [6]. Activation of the PI3/Akt and Erk-mediated pathways induces the survival and proliferation of cancer cells, eventually leading to TRAIL resistance [8]. Upregulation of cFLIP, a caspase-8 inhibitor [9] and mutations in the *Bax* and *Bak* genes [10] have been associated with decreased TRAIL-induced apoptosis in cancers. These findings have resulted in the development of promising TRAIL-sensitizing treatment strategies, including DR4/5 induction, Akt and Erk pathway inhibition, and the repression of cFLIP expression. Various anti-cancer drugs used in traditional chemotherapy, such as bortezomib, doxorubicin, valproic acids, or decitabin enhance the TRAIL sensitivity in the cancer cells, but these chemicals also exhibit cytotoxic effects in normal cells [11,12]. Therefore, there are continuing urgent needs to identify novel agents that can be used in combination with TRAIL to improve apoptotic efficacy and to overcome TRAIL resistance in cancer cells.

A number of studies have focused on oxidative agents which potentiate TRAIL-mediated apoptosis in a reactive oxygen species (ROS)-dependent manner [2,8,9,13,14,15,16]. These oxidative agents can promote diverse effects, such as inducing the upregulation of DR5, and promoting TRAIL-induced apoptosis [9,14,15,16]; also inhibiting oncogenic pathways and/or activating apoptotic pathways such as the NF-κB-mediated oncogenic signaling pathway and the ROS-mediated JNK-CHOP pathway [9,14,15,16]; furthermore, inducing ROS-dependent apoptosis via PTEN-mediated Akt inactivation and p53 activation [8]; and inducing cell membrane depolarization and disruption of intracellular ion homeostasis, possibly via impairment of ion channels or transporters for Na^+^, K^+^, Cl^−^, and Ca^2+^ [2].

Nonthermal (room temperature) plasma generated from microplasma jet devices is comprised of charged particles, some of which are reactive. Nonthermal plasma has recently emerged as a therapeutic agent for clinical applications such as in vivo antiseptics, wound healing, dermatology, dentistry, and cancer treatment. Such therapeutic applications have shaped the concept of plasma medicine. In previous studies, plasma was shown to efficiently induce apoptosis in cancer cells by disrupting mitochondrial membrane potentials and promoting mitochondrial ROS accumulation, consequently leading to ROS-dependent apoptotic cell death [17,18,19,20,21]. Moreover, plasma treatment does not significantly affect healthy cells [15,16,17,18,19]. So, it has been proposed that the level of plasma-generated ROS/RNS is high enough to induce cell death in cancer cells, but not in normal cells upon the same plasma-activated medium (PAM) treatment [17].

We investigated if PAM in combination with TRAIL (PAM/TRAIL sensitization) can induce apoptosis in TRAIL-resistant cancer cells. PAM/TRAIL sensitization has upregulated DR5 expression and membrane dysfunction, inducing ROS-dependent apoptosis of cancer cells. PAM/TRAIL sensitization could serve as a novel strategy to overcome TRAIL resistance in cancer cells. PAM is a promising candidate for further investigations as a chemotherapeutic sensitizer in the treatment of cancer.

## 2. Results

### 2.1. PAM Synergistically Enhances the Anticancer Efficacy of TRAIL

**Figure 1:**
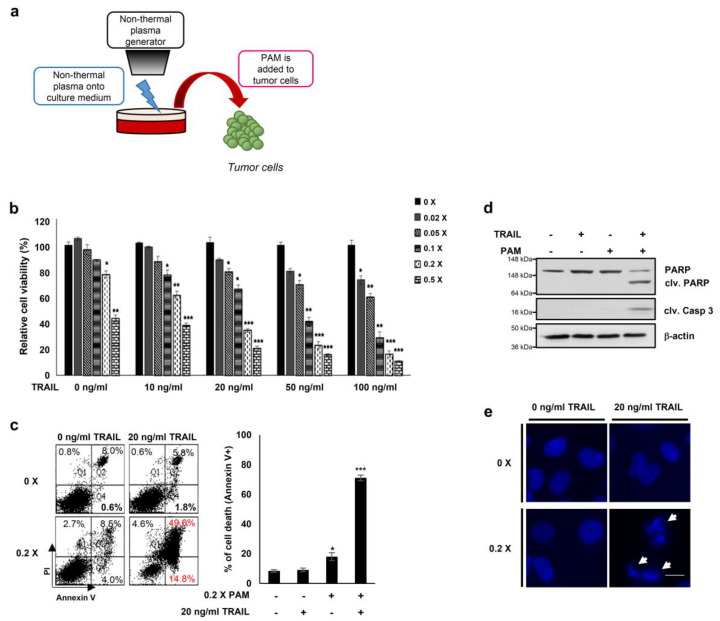
PAM and tumor necrosis factor-related apoptosis-inducing ligand (TRAIL) synergistically induce cancer cell death. (**a**) Preparation of plasma activated medium (PAM) using a non-thermal plasma generator system. PAM was generated by exposing DMEM or RPMI-1640 medium to non-thermal plasma jet at a distance of approximately 2 cm. (**b**) HeLa cells were treated with TRAIL at various concentrations (10 to 100 ng/mL) in the absence or presence of serially diluted PAM as indicated. Cell viability was assessed after 24 h of treatment using the MTT assay. * *p* < 0.05, ** *p* < 0.01, *** *p* < 0.001. (**c**) Cell death was analyzed using fluorescence-activated cell sorting (FACS) following Annexin V and propidium iodide (PI) staining. Left panel shows a representative image of necrosis and early and late apoptosis. Right panel shows statistical analysis of cell death. * *p* < 0.05, *** *p* < 0.001). (**d**) PAM promoted TRAIL-mediated apoptosis. Immunoblotting was performed using antibodies directed against cleaved caspase 3, PARP, and actin. (**e**) Condensation and fragmentation of the nuclei were detected via 4′,6-diamidino-2-phenylindole (DAPI) staining. Arrows: fragmented nuclei. Scale bar: 10 μm.

Previous reports have demonstrated that oxidative agents induce TRAIL sensitization [2] and that plasma mediates ROS-induced apoptosis of cancer cells [17,18,19,20,21]. Thus, to explore a new method for TRAIL sensitization, we generated PAM by spraying air plasma at atmospheric pressure onto the surface of DMEM media for 10 min (Figure 1a) [17,22]. We first determined levels of ROS (H_2_O_2_, hydrogen peroxide) and RNS (NO, nitrogen oxide) to be approximately 10 and 160 μM, respectively, in PAM (Supplementary Figure S1a,b). Next, we examined the TRAIL sensitizing effects of PAM in cervical cancer HeLa cells (Figure 1b–e). HeLa cells treated with either TRAIL alone (10–100 ng/mL) or with a 50-fold dilution of PAM alone did not affect cell viability (Figure 1b). The results of these experiments demonstrate that subtoxic doses of TRAIL and PAM are 10–100 ng/mL and 5- to 50-fold dilutions, respectively, when applied separately. However, HeLa cell viability was significantly reduced by treatment of PAM at various concentrations with a fixed TRAIL concentration or vice versa (Figure 1b). Flow cytometric analysis of annexin V/propidium iodide stained cells revealed that apoptosis was significantly induced in HeLa cells by co-treatment with a five-fold dilution of PAM and 20 ng/mL TRAIL for 24 h, but not with single treatment (Figure 1c). Furthermore, PAM/TRAIL treatment of HeLa cells significantly induced cleavage of the apoptotic markers such as caspase-3 and PARP (Figure 1d). DNA fragmentation was induced only by PAM/TRAIL treatment, but not by treatment with PAM or TRAIL separately (Figure 1e). These results demonstrate that PAM/TRAIL co-treatment of HeLa cells increases apoptosis.

### 2.2. PAM/TRAIL Treatment Induces Apoptosis in TRAIL-Resistant Cancer Cells but Not in Normal Cells

We investigated the cytotoxicity of PAM and/or TRAIL on multiple cell lines to determine if PAM can stimulate TRAIL-mediated apoptosis in TRAIL-resistant cancer cell lines. PAM/TRAIL treatment of HeLa, A549, or HepG2 cell lines resulted in cell growth inhibition and induction of cell death (Table 1 and Table 2, Supplementary Figure S2a,b). PAM/TRAIL treatment also reduced cell viability in the TRAIL-resistant DU145 cancer cell line (Supplementary Figure S3a). In contrast, the combined treatment did not significantly affect cell viability in the non-cancer human dermal fibroblast (HDF) cell line (Supplementary Figure S3a). The broad-spectrum caspase inhibitor zVAD abrogated apoptosis in cancer cells co-treated with PAM and TRAIL (Table 1 and Table 2, Supplementary Figure S2a,b), confirming that PAM/TRAIL treatment induces apoptosis in various TRAIL-resistant cancer cells but not in normal cells.

### 2.3. PAM/TRAIL Treatment Induces Apoptosis via DR5 Upregulation

**Figure 2:**
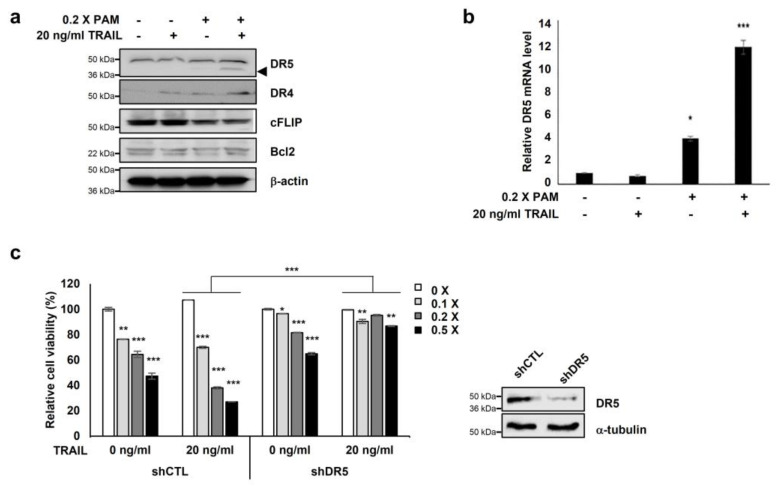
Co-treatment of PAM with TRAIL induces apoptosis via DR5 upregulation. (**a**) Combined treatment of PAM with TRAIL upregulates DR5 protein levels and downregulates c-FLIP protein levels in HeLa cells. Cells were treated with 0.2× PAM, TRAIL (20 ng/mL), or PAM/TRAIL (0.2× PAM with 20 ng/mL TRAIL). Cell extracts were prepared for immunoblotting of DR5, DR4, c-FLIP, Bcl-2, and β-actin. For immunoblotting, β-actin used as loading control. (**b**) DR5 mRNA levels were determined by qRT-PCR. GAPDH used as an internal control. (**c**) Silencing of *DR5* gene was abrogated synergistic effects of TRAIL to PAM-mediated cell survival inhibition. shCTL or shDR5-transfected HeLa cells were treated 0 or 20 ng/mL TRAIL with serial diluted PAM for 24 h. Cellular growth inhibition was determined via the MTT assay. * *p* < 0.05, ** *p* < 0.01, *** *p* < 0.001.

We investigated the expression of apoptosis-related proteins, Bcl-2 and c-FLIP, and the TRAIL receptors DR4 and DR5 to elucidate the molecular mechanisms underlying PAM-mediated TRAIL sensitization. PAM/TRAIL treatment increased DR4 levels and significantly increased DR5 levels but did not affect the expression levels of anti-apoptotic Bcl-2 proteins in HeLa cells (Figure 2a). Anti-apoptotic cFLIP protein levels were slightly decreased in PAM/TRAIL-treated cells (Figure 2a). We investigated the effects of PAM and PAM/TRAIL treatments on the transcriptional expressions of DR5, DR4, cFLIP, and Bcl-2. PAM/TRAIL treatment significantly increased DR5 mRNA compared to that of PAM alone (Figure 2b). However, contrast to DR5 (approximately 4–12 fold increase), mRNA levels of DR4, cFLIP, and Bcl-2 were not significantly different in treatments of PAM, TRAIL, or PAM/TRAIL combination in HeLa cells (Supplementary Figure S4). Also the DR5 transcription induced by PAM/TRAIL treatment was detected in A549 cells (Supplementary Figure S5), further validating that the combinational treatment of PAM and TRAIL upregulates DR5 transcription (Figure 2a,b).

To examine the functional role of DR5 in PAM/TRAIL-induced cytotoxicity, we tested the effects of the DR5-specific blocking chimeric antibody (DR5/Fc) and DR5 knockdown. Pretreatment with DR5/Fc (20 ng/mL) significantly reduced growth inhibition in PAM/TRAIL-treated A549, HeLa, HepG2, and DU145 cells by at least 45% (Table 1 and Supplementary Figure S3a). Also DR5/Fc pretreatment ameliorated the PAM/TRAIL-induced apoptosis in HeLa and A549 cells (Table 2 and Supplementary Figure S3b). Knockdown of DR5 protected HeLa cells from cell death induced by PAM/TRAIL treatment (Figure 2c). These results demonstrate that DR5 upregulation is required for PAM/TRAIL-induced apoptosis.

### 2.4. CHOP Mediates DR5 Upregulation Induced by PAM/TRAIL Treatment

**Figure 3:**
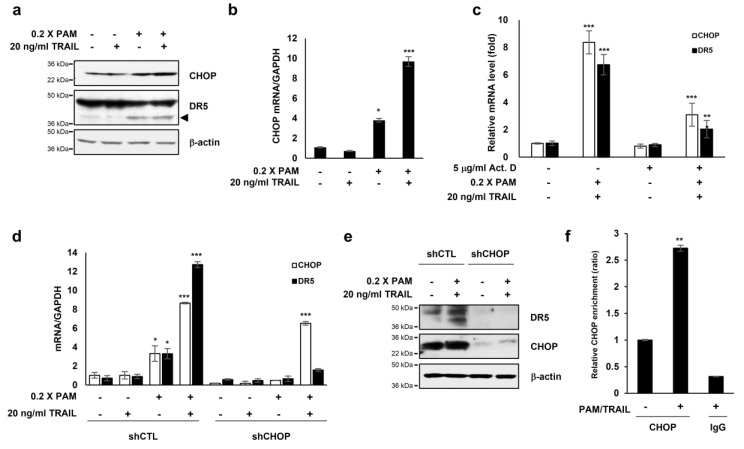
PAM/TRAIL induces DR5 upregulation through CHOP-mediated transcription. (**a**) HeLa cells were treated with 0.2× PAM alone or PAM/TRAIL. After 24 h, proteins in cell lysates were separated by sodium dodecyl sulfate polyacrylamide gel electrophoresis and immunoblotting with antibodies against CHOP, DR5, and β-actin. (**b**) HeLa cells were treated as described in (**a**). After 24 h, CHOP mRNA levels were determined via qRT-PCR. GAPDH was used as an internal control. (**c**) HeLa cells were co-treated with PAM/TRAIL for 12 h. Afterwards, the culture was replaced with fresh medium, pretreated with or without 5 μg/mL actinomycin D (Act D) for 30 min, and co-treated with or without PAM/TRAIL for 10 h. mRNA levels of CHOP and DR5 were determined via qPCR. (**d**,**e**) HeLa cells were transfected with a plasmid expressing either CHOP shRNA (shCHOP) or control shRNA (shCTL). At 24 h after transfection, cells were treated with PAM, TRAIL, or PAM/TRAIL for an additional 24 h. (**d**) Levels of CHOP and DR5 mRNA were determined via qRT-PCR. (**e**) Protein levels of CHOP and DR5 were determined by immunoblot analysis using antibodies against DR5 and CHOP. (**f**) Chromatin immunoprecipitation (ChIP) was performed using anti-CHOP antibody in HeLa cells following PAM/TRAIL treatment for 8 h. The IgG was used to control for antibody specificity. qRT-PCR was carried out using primers surrounding the CHOP binding sites in the DR5 promoter. * *p* < 0.05, ** *p* < 0.01, *** *p* < 0.001.

The transcription factor CCAAT/enhancer binding protein (C/EBP) homologous protein (CHOP) is known to activate DR5 transcription [23] resulting in TRAIL sensitization in various cancer cells [16,24]. Our investigations showed that PAM or PAM/TRAIL treatment induced CHOP expression at both protein and mRNA levels (Figure 3a,b), concomitant with the increase in transcriptional and protein expressions of DR5 (Figure 2a,b). We investigated if treatment with PAM/TRAIL upregulates CHOP expression, which in turn increases DR5 expression. Actinomycin D abrogated PAM/TRAIL-induced up-regulation of both CHOP and DR5 transcriptions (Figure 3c). We used CHOP knockdown to investigate the relationship between PAM/TRAIL treatment and DR5 mRNA upregulation. In the absence of CHOP, PAM/TRAIL treatment did not increase expression of DR5 mRNA or protein (Figure 3d,e). To see whether CHOP is implicated in the PAM/TRAIL-induced DR5 transcription, we performed ChIP-qPCR assay. CHOP was recruited at the DR5 promoter (−276 to −264) [25] in the presence of PAM/TRAIL (Figure 3f). These results demonstrate that PAM/TRAIL treatment induces CHOP-mediated upregulation of DR5 expression.

### 2.5. Plasma-Activated Medium (PAM) Promotes Membrane-Bound DR5 Redistribution

**Figure 4:**
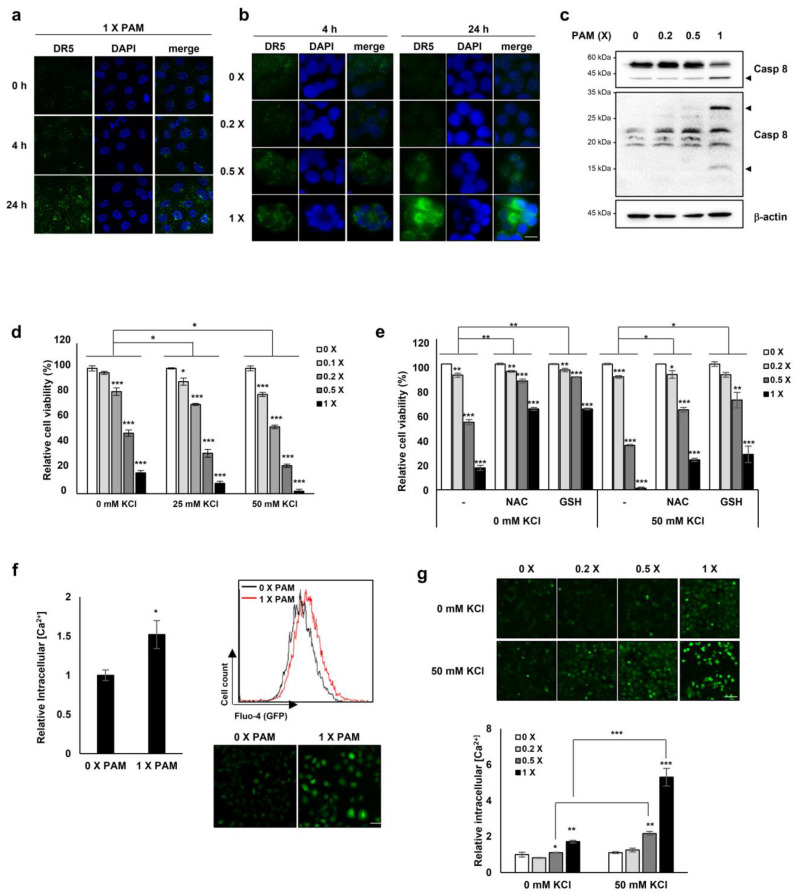
PAM promotes membrane-bound DR5 redistribution. HeLa cervical cancer cells (**a**) and HT-29 colorectal cancer cells (**b**) were treated with serially diluted PAM for 4 or 24 h. Original magnification: × 630. Scale bar: 10 μm. After PAM treatment, cells were fixed with 4% paraformaldehyde for 15 min followed by immunostaining with anti-DR5 antibody. DR5 clustering was detected by fluorescence microscopy. (**c**) HeLa cells were treated with PAM for 24 h, as indicated. Caspase 8 protein levels were determined by immunoblotting. β-actin was used as a loading control. Arrow head: cleaved form of caspase 8. (**d**) The effects of PAM on A549 cancer cells. A549 lung cancer cells were treated with serially diluted PAM supplemented with 0, 25, or 50 mM KCl for 24 h. Cell viability was measured using the MTT assay. (**e**) The antioxidants, N-acetyl-cysteine (NAC), and reduced glutathione (GSH) abrogate the impaired ion homeostasis induced by PAM. A549 cells were treated with serially diluted PAM in the absence or presence of 50 mM KCl supplemented with 2 mM NAC or 1 mM GSH for 24 h. (**f**) Intracellular concentration of Ca^2+^ was measured following PAM treatment of A549 cells. After incubation with 2 μM Fluo-4, AM for 45 min, intracellular Ca^2+^concentration was analyzed by FACS analysis or by fluorescence microscopy. Scale bar: 50 μm. (**g**) U2OS cells were treated with serially diluted PAM in the absence or presence of 50 mM KCl for 24 h, and then the level of intracellular Ca^2+^ was evaluated. * *p* < 0.05, ** *p* < 0.01, *** *p* < 0.001. Scale bar: 50 μm.

Next we investigated the effect of PAM treatment on the expression and clustering of DR5 on the membrane, similarly to the case of the membrane-bound death receptor Fas whose forced redistribution and aggregation into ceramide-rich lipid platforms enhance FasL-mediated apoptosis [26]. Undiluted and 0.5× PAM enhanced the membrane clustering of DR5 in HeLa and HT-29 cells, inducing its redistribution in a dose- and time-dependent manner (Figure 4a,b).

Because the ligand TRAIL is required for DR5 membrane clustering [27] and we showed that the lethal-dose (undiluted and 0.5×) of PAM promoted DR5 clustering on the membrane (Figure 4a,b), we examined whether PAM could induce TRAIL expression. We evaluated the TRAIL mRNA levels with and without PAM treatment. The TRAIL mRNA levels were barely affected by the sublethal- (0.2×) and lethal (0.5×) doses of PAM (Supplementary Figure S6), indicating that the PAM-mediated DR5 redistribution on the membrane may occur in a TRAIL-independent manner. Since a previous study showed that up-regulated DR5 induces TRAIL-independent apoptosis via caspase 8 [28], we examined whether the overexpressed DR5 by PAM (Figure 4a,b) induces caspase 8-dependent apoptosis. In the 1 × PAM-treated HeLa cells, cleaved caspase 8 was increased (Figure 4c), suggesting that the PAM-DR5-caspase 8 axis is distinct from TRAIL-induced apoptosis.

The observation of PAM-induced DR5 redistribution on the membrane (Figure 4a,b), led us to question whether PAM may induce membrane alternations, also. Previously we reported that plasma-induced ROS/RNS disturbs mitochondrial membrane potential [19] and impairs cellular membrane through coincident lipid oxidation, altered electrical conductivity, and membrane roughening [20]. Impairment of cellular membranes [20] could compromise the maintenance of transmembrane ion gradients, rendering cells vulnerable to extracellular ion stress. We investigated cell viability with and without PAM under conditions of high [K^+^], a membrane depolarizing agent. PAM increased cell death as [K^+^] increased (Figure 4d). The addition of the antioxidants glutathione or N-acetyl-cysteine (NAC) significantly reduced PAM-induced cell death under conditions of high [K^+^] (Figure 4e), indicating that PAM-induced oxidative stress contributes to cell death. We assessed intracellular Ca^2+^ concentrations in the presence or absence of PAM to investigate if PAM-induced cellular membrane damage might disrupt ion homeostasis. Exposure of A549 cells to PAM resulted in an elevation of Ca^2+^ by approximately 1.5-fold compared to untreated cells (Figure 4f). PAM-induced accumulation of intracellular Ca^2+^ was synergistically enhanced in U2OS cells in the presence of 50 mM KCl (Figure 4g). These results indicate that PAM compromises cellular membranes, disrupting intracellular Ca^2+^ ion homeostasis.

### 2.6. ROS is Implicated in PAM/TRAIL Sensitization

**Figure 5:**
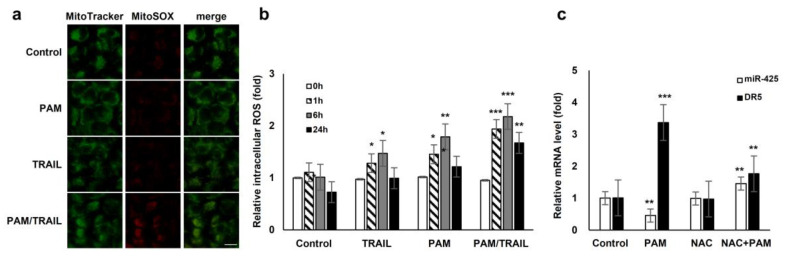
ROS generation is involved in PAM-mediated TRAIL sensitization. (**a**) Reactive oxygen species (ROS) production in mitochondria was induced by treatment with PAM/TRAIL. HeLa cells were treated with PAM, TRAIL, or PAM/TRAIL, after which mitochondrial ROS accumulation was assessed after 24 h by staining the cells with MitoTracker Green and MitoSox Red under a fluorescent microscope. Scale bar: 10 μm. (**b**) Intracellular ROS generation was induced by treatment with PAM/TRAIL. Cells were treated as in (**a**) for the indicated times. Intracellular ROS were quantified via the H_2_DCF-DA assay. * *p* < 0.05, ** *p* < 0.01, *** *p* < 0.001. (**c**) PAM-induced ROS generation is required for DR5 upregulation and miR-425 downregulation. Pretreatment with a surrogate antioxidant, NAC, reversed PAM-induced DR5, and miR-425 transcriptional modulation. ** *p* < 0.01, *** *p* < 0.001.

We investigated the involvement of PAM/TRAIL-sensitization in the course of intracellular ROS production. PAM/TRAIL treatment revealed a significant increase in intracellular ROS levels in HeLa cells as determined by MitoSOX-based fluorescent microscopy (Figure 5a) and H_2_DCFDA-based FACS analysis (Figure 5b). We next investigated the role of ROS in PAM-induced DR5 mRNA expression, as PAM induced the DR5 expression levels (Figure 2a,b). Pretreatment of HeLa cells with the antioxidant NAC abrogated PAM-induced DR5 transcription (Figure 5c), indicating that PAM-induced ROS is involved in DR5 mRNA upregulation. Furthermore, NAC pretreatment attenuated PAM/TRAIL-induced growth inhibition and cell death in cancer cells, including HeLa, A549, and HepG2 (Table 1 and Table 2, Supplementary Figure S7). Because PAM/TRAIL treatment induced generation of ROS that could be originated from mitochondria (Figure 5a), we investigated the consequence of mitochondrial ROS inhibition in the presence of PAM/TRAIL treatment. MnTBAP and Ebselen, mimetic agents for superoxide dismutase (SOD) and glutathione peroxidase, respectively, abrogated PAM/TRAIL-induced cytotoxic effects in HeLa cells (Supplementary Figure S8). These results indicate that DR5 upregulation and TRAIL sensitization are dependent on PAM-induced ROS generation.

### 2.7. PAM Sensitizes Cancer Cells to TRAIL-Induced Apoptosis via Modulation of miR-425-PTEN-Akt Axis

**Figure 6:**
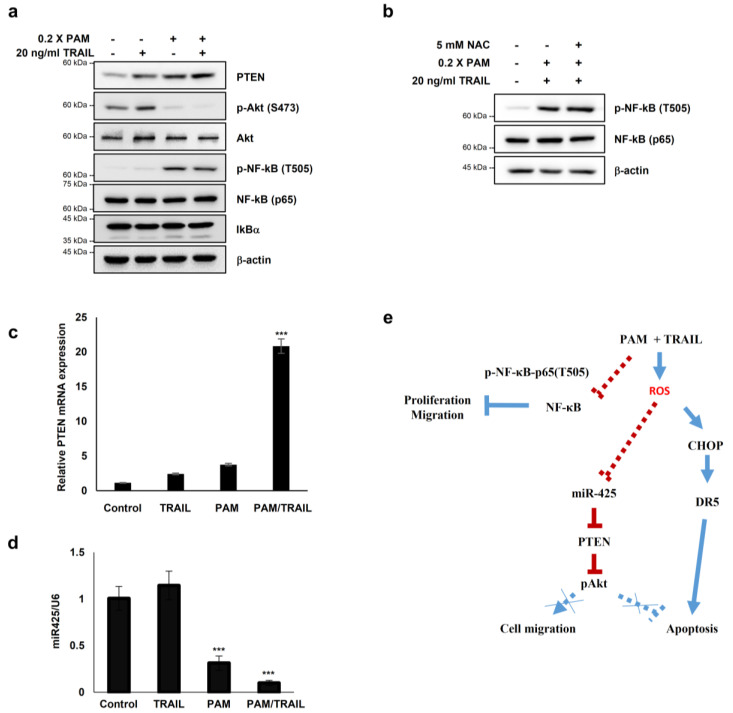
PAM sensitizes cancer cells to TRAIL-induced apoptosis via modulation of the miR-425-PTEN-Akt axis. (**a**–**d**) HeLa cells were treated with TRAIL with or without PAM for 24 h. (**a**) PTEN, phospho-Akt1 (S473), Akt1, NF-κB, phospho-NF-κB (T505) and IκBα protein levels were determined by immunoblotting. β-actin was used as a loading control. (**b**) HeLa cells were pretreated with 5 mM NAC for 1 h before TRAIL treatment in the presence of PAM. After 24 h, phosphor-NF-κB (T505) and NF-κB protein levels were observed by immunoblot analysis. (**c**) PTEN mRNA levels were determined by qRT-PCR. *** *p* < 0.001. (**d**) miR-425 levels were determined via qRT-PCR. *** *p* < 0.001. (**e**) Proposed model for PAM-mediated TRAIL sensitization. Our work has revealed that combinational treatment with PAM and TRAIL stimulates ROS-dependent apoptosis by two attributes; (1) via CHOP-mediated upregulation of DR5 transcription (solid blue line), and (2) via miR-425 suppression-mediated PTEN upregulation, resulting in Akt inactivation (solid red line). In this study, PAM and TRAIL cotreatment induces inhibitory phosphorylation of NF-κB (solid red line). Previous studies have provided evidences that active NF-κB and phospho-Akt promote proliferation and migration (dotted blue lines).

Several studies have reported that Akt inactivation induces TRAIL sensitization in TRAIL-resistant cancers [13]. We investigated the involvement of PTEN-Akt signaling in PAM/TRAIL-induced apoptosis. Following PAM or PAM/TRAIL treatment, levels of PTEN protein, an upstream phosphatase of Akt signal pathway, were markedly increased, and accordingly phosphorylated Akt levels were decreased in HeLa cells (Figure 6a). These results demonstrate that PAM-induced PTEN upregulation, leading to Akt inactivation, contributes to TRAIL-mediated apoptosis by PAM.

In order to determine whether PAM- and PAM/TRAIL-induced PTEN expression (Figure 6a) occurs at the transcriptional level, we evaluated the mRNA levels of PTEN in the PAM and TRAIL treated HeLa cells. PAM- and PAM/TRAIL-treatment increased PTEN mRNA levels, to approximately 4- and 21-fold, respectively (Figure 6c). Based on the previous studies demonstrating that miR-425-mediated suppression of PTEN promotes cancer aggressiveness and is associated with tumorigenesis and malignancy in many cancers [29,30,31], we tested whether PAM- and PAM/TRAIL-treatment can downregulate the miR-425 transcription. The levels of miR-425 significantly decreased in PAM- and PAM/TRAIL-treated HeLa cells (Figure 6d), concomitantly with the increased PTEN mRNA (Figure 6c). PAM-mediated suppression of miR-425 was abrogated by NAC (Figure 5c) indicating that downregulation of miR-425 is possibly mediated through PAM-induced ROS generation, similar to NAC ablation of PAM-induced DR5 upregulation. In order to determine whether suppression of miR-425 mediates PTEN transcription in response to PAM or PAM/TRAIL treatment, we investigated levels of PTEN protein in miR-425-transfeccted HeLa cells to confirm the transcription response of PTEN to miR-425. The increase in PTEN protein by PAM or PAM/TRAIL treatment was reduced in miR-425-transfected HeLa cells (Supplementary Figure S9a). PAM treatment induced the downregulation of miR-425 and the increase in TRAIL sensitization in different TRAIL-resistant cell lines, including DU145, U87-MG, MCF7, and MDA-MB-435 (Supplementary Figure S9b). These results show that PAM/TRAIL treatment can enhance PTEN transcription through suppression of miR-425, increasing PTEN protein levels, leading to Akt inactivation and apoptosis.

Furthermore, one of the well-known regulatory mechanisms for tumor cells is phosphorylation of NF-κB RelA (p65) at T505, which mediates cellular apoptosis and inhibits proliferation and migration through differential gene regulation [31]. Hence, we tested whether PAM- and PAM/TRAIL-treatment regulates phosphorylation of p65 at T505. The phospho-p65 at T505 was induced by PAM- and PAM/TRAIL treatment (Figure 6a). Next, we examined whether the phosphorylation of p65 at T505 is regulated by activation or translocation of NF-κB. The protein levels of IκBα, an inhibitory regulator of NF-κB was not altered by treatment of PAM/TRAIL (Figure 6a), suggesting that the phosphorylation of p65 at T505 is not relevant to the canonical NF-κB activation. Together, these results suggest that PAM/TRAIL-induced p65 phosphorylation may contribute to cancer cell apoptosis.

To investigate whether the PAM-induced p65 phosphorylation associated with NF-κB inhibition depends on ROS generated in PAM, we evaluated the level of p65 phosphorylation at T505 in the NAC-pretreated HeLa cells prior to PAM/TRAIL-treatment. The level of phospho-p65 (T505) was not affected by NAC (Figure 6b), suggesting that the inhibitory phosphorylation of p65 may not depend on the ROS generated in PAM.

## 3. Discussion

The selective induction of apoptosis by TRAIL and agonistic TRAIL receptor antibodies in cancer cells has led to their clinical development as promising anticancer therapeutics. However, in many cancers, TRAIL resistance is intrinsic or acquired during the course of TRAIL treatment [8,9]. To overcome this resistance, it is important to identify effective TRAIL sensitizers that target tumor heterogeneity in the TRAIL pathway for patient-tailored therapy [32]. In this study, we explored the ability of PAM to potentiate TRAIL-induced ROS-mediated apoptosis of cancer cells. We investigated the effects of PAM on TRAIL sensitization and the molecular mechanisms behind TRAIL-resistant A549 and HeLa cancer cells. PAM triggers ROS/RNS generation, leading to transcriptional upregulation of DR5 via CHOP-mediated transcriptional activation, disruption of intracellular Ca^2+^ homeostasis, and inactivation of Akt via PTEN upregulation. The role of PAM/TRAIL sensitization, and its underlying mechanisms involving the generation of ROS/RNS [21] is further supported by results showing that antioxidants, such as NAC and glutathione which can prevent cellular accumulation of ROS/RNS [21], reduce TRAIL sensitization, mitigate DR5 upregulation, and reduce intracellular Ca^2+^ aberration.

PAM has potent anti-cancer activity because of its reactive oxygen and nitrogen species and is effective regardless of tumor genetic variations [21]. In recent years, plasma has been used in fields such as medical disinfection and antiseptics, blood clotting, dentistry, and skin care. Its use is steadily increased in immunotherapy, wound regeneration, and cancer treatment. Accumulating experimental and animal evidence demonstrates plasma is a safe medicinal agent that does not have adverse effects on healthy normal cells [17,18,21]. Recent studies have shown that plasma exhibits anticancer efficacy against a broad range of cancers and that PAM addresses the problem of limited penetrance in cell culture studies and rodent models [17,18,21]. Here, we show that PAM/TRAIL treatment induces apoptosis in TRAIL-resistant cancer cells without side effects on normal cells.

Our current findings demonstrate that CHOP induction by PAM induces transcriptional activation of DR5, leading to DR5 upregulation and further induction of TRAIL-mediated apoptosis. Incubation of cells with antibodies against DR5 attenuated PAM/TRAIL-induced reduction in cell viability, indicating that PAM is capable of provoking apoptosis in TRAIL-resistant cancer cells. Our results show that PAM induced the expression of the DR5 transcriptional activator, CHOP, resulting in increasing DR5 expression. In cells lacking CHOP, PAM-induced DR5 upregulation and TRAIL-induced apoptosis were reduced, confirming that DR5 upregulation by PAM is mediated through CHOP induction. Our results are consistent with previous findings indicating that CHOP is required for PAM-induced DR5 transcriptional upregulation [16,23,33].

The oxidative stress-dependent effects of PAM on DR5 clustering (Figure 4a,b) and membrane dysfunction (Figure 4d) [19,20,21] are similar to interaction mechanisms between oxidative stress and TRAIL-induced apoptosis [2,8,9,15]. PAM elevates intracellular Ca^2+^ levels possibly through impairment of ion channel or transporters and permeability transitions. PAM-induced membrane dysfunction was enhanced under high-salt (KCl) stress, indicating a lack of membrane integrity (Figure 4d). K^+^ potentiates TRAIL-induced apoptosis in human tumor cells, including leukemia, melanoma, and lung cancer cells via mitochondria-derived ROS (mROS) accumulation [34]. Inactivation of Na^+^-K^+^-ATPase and sustained membrane depolarization were observed during Fas-induced apoptosis [35,36], initiated by intracellular glutathione depletion and H_2_O_2_ generation [37]. ROS have been shown to stimulate external Ca^2+^ entry into the cytoplasm, leading to the generation of intracellular mROS and mitochondrial damage [38,39]. Mitochondrial damage induces a release of cytochrome c and other apoptotic protein factors that enhance necrosis and apoptotic cell death [36,40]. Depolarization can act in both pro- and anti-apoptotic processes, depending on the cell types and apoptotic stimuli involved. Suzuki Y et al. reported that the depolarization appears to be a prerequisite event for TRAIL-induced apoptosis, because TRAIL induces minimal cytotoxicity despite the substantial expression of DR4 and DR5 in the cellular membrane [41]. Our results indicate that PAM first induces ROS-mediated membrane depolarization followed by DR5 clustering and TRAIL-induced apoptosis.

TRAIL-induced DR4 or DR5 trimerization activates the extrinsic apoptotic pathway [42,43,44]. Once trimerized, DR4 or DR5 serves as the aggregation point for a multimeric protein structure called “death-induced signaling complex (DISC)” that is comprised of the ligated DR4/5, Fas-associated death-domain protein (FADD), and procaspases 8 and 10. Caspases play essential roles in programmed cell death including apoptosis and necroptosis. When the caspase 8 activity is inhibited by zVAD, necroptosis is initiated [45]. Also, numerous necroptosis-insensitive cancer cell lines, such as HeLa, HCT116, and OVCAR4 (human ovarian cancer) cells do not have an effective necroptotic machinery. The necroptosis-sensitive cells, such as A549 and HepG2, are responsive to a few specific necroptosis inducers, but not to all necroptosis inducers, revealing that necroptosis can occur by specific stress signals in a controlled manner [45]. Therefore, we tested whether the growth inhibition by PAM/TRAIL treatment was caused by necroptosis as well as apoptosis. As shown in Supplementary Figure S2c, necrostatin-1(Nec-1, an inhibitor of necroptosis) had little effects on the PAM/TRAIL-induced growth inhibition in the both TRAIL-unresponsive HeLa (necroptotic-insensitive) and A549 (necroptotic-sensitive) cells. Also phosphorylation of RIP3 and MLKL (necroptosis markers) were not changed by treatment of zVAD or Nec-1 in the PAM/TRAIL-treated HeLa cells (Supplementary Figure S2d). In the Table 1 and Supplementary Figure S2, the inhibitory effect of zVAD on the PAM/TRAIL-mediated cell death in HepG2 cells was slightly lower than that in HeLa and A549 cells, suggesting that PAM/TRAIL may induce necroptosis in HepG2 cells. Additionally, DR5/Fc inhibited more effectively the PAM/TRAIL-mediated cell death in HeLa and A549 cells, compared with HepG2 cells. Taken together, these data indicate that these inhibitors (zVAD, Nec-1, and DR5/Fc) have differential effects depending on the type of cell lines. However, the inhibitory effects of NAC on the PAM/TRAIL-mediated cell death were comparable in the three tested cell lines, supporting that the PAM/TRAIL-mediated cell death occur in an ROS-dependent manner, in the tested cell lines.

The microRNAs miR-221, -222, and -425 are known to target PTEN and induce TRAIL resistance in cancer cells [30,33]. Here, we found that PAM treatment leads to upregulation of PTEN and reduction of phosphorylated Akt levels, eventually sensitizing cells to TRAIL-induced apoptosis through inactivation of the Akt pathway. Consistent with earlier studies [30,33], PAM treatment suppressed miR-425 expression, resulting in increased PTEN expression and leading to TRAIL-inducing apoptosis mediated by Akt inactivation. In addition, PAM treatment decreased the expression of the cell survival protein c-FLIP, which is linked to TRAIL resistance.

The NF-κB pathway contributes to the growth, survival, and malignancy of numerous cancer cell types while also affecting the response of tumors to chemotherapy and radiotherapy [46]. Post-translational modifications are crucial for the functions of NF-κB subunits [47], such as phosphorylations of p65 at T254, S276, S281, S316, S468 lead to cell proliferation, migration, and ubiquitination [48,49,50,51,52]. In contrast, threonine505 (T505) residue in p65 with an important inhibitory regulation role is phosphorylated by Chk1 in response to cisplatin. The inhibitory p65 phosphorylation at T505 leads to induction of the tumor suppressor p14ARF, which contributes to tumor suppression [53]. We found that phosphorylation of NF-κB at T505 was increased by PAM or PAM/TRAIL treatment (Figure 6a), suggesting the possibility that PAM or PAM/TRAIL may suppress migration and proliferation of cancer cells via inhibition of NF-κB signal pathway.

Consistent with prior studies, we found that PAM treatment induced the generation of intracellular and mitochondrial ROS, which likely mediates the upregulation of DR5 and sensitizes cells to TRAIL-induced apoptosis [9,54,55,56]. We found that the antioxidant NAC significantly ameliorated PAM-induced TRAIL-mediated apoptosis with concomitant recovery of Ca^2+^ homeostasis and the abolishment of DR5 upregulation, confirming that ROS/RNS plays a crucial role in PAM-induced TRAIL sensitization. Our results demonstrate that PAM induces ROS/RNS production, which in turn leads to CHOP-induced DR5 upregulation, and consequently gives rise to the TRAIL-induced apoptosis of cancer cells.

In summary, PAM may be beneficial for sensitization of TRAIL-resistant cancer cells leading to apoptosis. Clinically, resistance to anticancer drugs is one of major causes in treatment failure due to primary or acquired non-responsiveness of cancer cells. We demonstrated that various cancer cells including TRAIL resistant cells can be sensitized by PAM, while PAM has no cytotoxic effects on non-cancerous cells. Moreover, in xenograft formation using A549 adenocarcinoma in the mouse model, the anticancer effect of PAM was detected by assessing tumor volume. Thus, further studies to verify anticancer effects of PAM/TRAIL using in vivo tumor model have important implications for the development of novel strategies in cancer therapy.

## 4. Materials and Methods

### 4.1. Reagents and Antibodies

The following reagents were used in the experiments: recombinant protein human TRAIL/Apo2L (KOMA Biotech, Seoul, Korea); FITC Annexin V Apoptosis Kit I (BD Pharmingen, Franklin Lakes, NJ, USA); z-VAD-fmk (Promega, Madison, WI, USA); N-acetyl-cysteine, reduced glutathione, Mn(III) tetrakis (4-benzoic acid) porphyrin chloride (MnTBAP), Ebselen, necrostatin-1 (Sigma Aldrich, St. Louis, MO, USA); MitoTracker™ Green FM, MitoSOX™ Red, Fluo-4 AM, 2′,7′-dichlorodihydrofluorescein diacetate (H_2_DCFDA; Invitrogen, Carlsbad, CA, USA). We used antibodies against β-actin, IκBα (Santa Cruz Biotechnology, Inc., Dallas, TX, USA), DR4, phospho-RIP3 (T231/S232), phospho-MLKL (Abcam, Cambridge, UK), CHOP, caspase-3, caspase-8, p65 (Cell Signaling Technology, Danvers, MA, USA), poly(ADP-ribose) polymerase (BD Pharmingen), DR5 (KOMA Biotech and Abcam), α-tubulin (Millipore, Burlington, MA, USA), c-FLIP (Alexis, Farmingdale, NY, USA), phospho-p65 (T505) (Bioss antibodies, Woburn, MA, USA), and horseradish peroxidase (HRP)-conjugated anti-rabbit or anti-mouse IgG antibodies (Enzo Life Sciences, Farmingdale, NY, USA).

### 4.2. Immunostaining of DR5

HeLa and HT-29 cells were treated with serially diluted PAM for 4 or 24 h. After treatment, cells were fixed for 15 min with 4% paraformaldehyde and blocked in PBS containing 10% FBS for 30 min. Cells were incubated with antibody against DR5 (Abcam) for 16 h at 4 °C and then with FITC-conjugated secondary antibodies (Invitrogen) for 1 h at room temperature. After counterstaining with 4′6-diamidino-2-phenylindole (DAPI), immunofluorescence images were acquired using a fluorescence microscope (Eclipse Ti-S, Nikon Instruments Inc., Walt Whitman Road Melville, NY, USA).

### 4.3. Cell Culture and Treatment with Plasma-Activated Medium (PAM) and TRAIL

HeLa, A549, HepG2, U2OS, and HDF cells were obtained from American Type Culture Collection (ATCC) and cultured in DMEM (WELGENE, Kyung-san, Korea) supplemented with 10% fetal bovine serum (FBS) and antibiotics (Life Technologies, Carlsbad, CA, USA). HCT116 and HT-29 cells were cultured in RPMI-1640 containing 10% FBS and antibiotics. Plasma-activated medium (PAM) was produced using a microplasma jet device at atmospheric pressure [17,18,43] and discharging the plasma jet onto a liquid such as the mammalian cell culture medium DMEM (Figure 1a) [21]. Using the air plasma-jet system (AMED, Seoul, Korea), we jetted non-thermal air plasma 2 cm above the surface of growth medium, in a chamber of a 12-well-plate for 10 min at atmospheric pressure and room temperature to generate PAM [22]. Cells were washed with DPBS (Life Technologies) and covered with fresh culture medium that was either untreated or treated with undiluted or diluted plasma-activated medium (PAM) as indicated [21,43]. TRAIL was added to the culture medium at the indicated concentrations, either alone or in combination with PAM. Detection of apoptosis and the MTT colorimetric assay were performed as previously described [17,18,21,22].

### 4.4. Detection of Nuclei Condensation and Fragmentation

Cells were fixed with 1% paraformaldehyde, followed by staining with 300 nM DAPI for 5 min. Morphology of nuclei were observed under a fluorescence microscope (Eclipse Ti-S, Nikon Instruments Inc., Walt Whitman Road Melville, NY, USA).

### 4.5. Quantification of Mitochondrial and Intracellular ROS

Mitochondrial and intracellular ROS measurements were performed as previously described [17] according to the manufacturer’s protocol. Relative ROS levels were expressed as arbitrary fluorescence units.

### 4.6. Quantification of ROS and RNS of PAM

Concentration of H_2_O_2_ in PAM was measured using the Amplex Red Hydrogen Peroxide/Peroxidase Assay Kit (Invitrogen) and colorimetric intensity was measured on a microplate reader (Bio-Rad, Hercules, CA, USA) at 540/595 nm, according to the manufacturer’s protocol. Production of RNS in PAM was measured by the Griess assay [57].

### 4.7. Measurement of Intracellular Ca^2+^

A549 and U2OS cells were treated with PAM for 24 h, followed by staining with 2 μM Fluo-4 AM for 45 min. Cells were harvested by trypsinization and suspension in PBS. Intracellular Ca^2+^ was analyzed by flow cytometry and fluorescence microscopy.

### 4.8. RNA Isolation and Quantitative Real-Time PCR (qRT-PCR)

Total RNA was isolated using the RNeasy Min Kit (Qiagen, Carlsbad, CA, USA), and cDNA was synthesized using M-MLV reverse transcriptase (Promega) according to the manufacturer’s instructions. The following primers were used for amplification of human DR5, PTEN, DR4, cFLIP, Bcl-2, TRAIL, and GAPDH: DR5 (sense): 5′-GTC ACA GTT GCA GCC GTA GT-3′ and (antisense) 5′-TGC CTT TCA GGT AAG GAA GG-3′; PTEN (sense): 5′-GAT GTG GCG GGA CTC TTT AT-3′ and (antisense): 5′-AGC GGC TCA ACT CTC AAA CT-3′; DR4 (sense): 5′-AGA GAG AAG TCC CTG CAC CA-3′ and (antisense): 5′-GTC ACT CCA GGG CGT ACA AT-3′; cFLIP (sense): 5′-GCA AGA CCC TTG TGA GCT TC-3′ and (antisense): 5′-TCG CCT CAC TCT GTA GAG CA-3′; Bcl-2 (sense): 5′-GAG GAT TGT GGC CTT CTT TG-3′ and (antisense): 5′-ACA GTT CCA CAA AGG CAT CC-3′; TRAIL (sense): 5′-GGA ACC CAA GGT GGG TAG AT-3′ and (antisense): 5′-TCT CAC CAC ACT GCA ACC TC-3′; GAPDH (sense): 5′-GTC AAC GGA TTT GGT CTG TAT T-3′ and (antisense) 5′-AGT CTT CTG GGT GGC AGT GAT-3′. The following primers were used for amplification of miR-425: Specific primer: 5′-TGG ACC AGA ATG ACA CGA TCA CTC C-3′ and Universal reverse primer: 5′-GTG CAG GGT CCG AGG T-3′. Real-time PCR amplification was carried out using Qiagen Rotor-Gene Q system with the following cycling conditions: 95 °C for 10 min; 40 cycles of 95 °C for 15 s, 60 °C for 30 s, and 72 °C for 30 s; and a final extension step at 72 °C for 10 min.

### 4.9. Immunoblotting

Immunoblotting experiments were performed as previously described [19]. Representative results from at least three independent experiments are shown in the figures.

### 4.10. Plasmid Construction and Transfection

Double-stranded small interfering RNA (siRNA) against CHOP was generated using a pSUPER.retro.puro, an H1 promoter-driven RNA interference retroviral vector (Oligoengine, Seattle, WA, USA). The siRNA was designed to target CHOP (5′-GAT CGA CGT GTA GTG AAT G-3′) and DR5 (5′-GAC CCT TGT GCT CGT TGT C-3′). The miR-425 (5′-AAU GAC ACG AUC ACU CCC GUU GA-3′) was synthesized by Geneolution, Inc. (Seoul, Korea). Transfections were performed using the Effectene Kit (Qiagen) and Lipofectamine 2000 (Invitrogen) using manufacture’s manual.

### 4.11. Chromatin Immunoprecipitation (ChIP) Assay

ChIP assay with CHOP antibody (Cell signaling) was performed using the Chromatin Immunoprecipitation Assay Kit (Millipore) according to the manufacturer’s manual. The precipitates were analyzed by qRT-PCR using the primers 5′-AGG TTA GTT CCG GTC CCT TC-3′ and 5′-CAA CTG CAA ATT CCA CCA CA-3′ to amplify a DR5 promoter fragment containing CHOP binding site (−276 to −264).

### 4.12. Statistical Analysis

All data were expressed as mean ± standard deviation (SD) of at least three replicates. The Student’s *t*-test was used for comparisons between two groups. One-way or two-way ANOVA analysis followed by post-hoc test was used for comparisons between multiple groups. Differences were considered statistically significant at *p* < 0.05 (in figures: * *p* < 0.05, ** *p* < 0.01, *** *p* < 0.001).

## 5. Conclusions

Our results provide evidence that PAM synergistically enhances the efficacy of TRAIL-induced apoptosis in TRAIL-resistant cells by triggering ROS/RNS generation, which in turn upregulates DR5 expression via CHOP-mediated transcription, disturbs Ca^2+^ homeostasis, and inactivates Akt-mediated TRAIL resistance via suppression of miRNAs targeting PTEN. The findings that the oxidative stress-dependent PAM effects recapitulate the physiological condition in TRAIL-sensitive cells importantly provide a molecular basis for TRAIL sensitization. Taken together, these data highlight an oxidative stress-mediated mechanism through which CHOP-mediated DR5 upregulation, disturbance of ion homeostasis, and miRNA-mediated PTEN upregulation could promote TRAIL-induced apoptosis (Figure 6e). Thus, co-treatment with PAM and TRAIL serves as a novel combinational therapeutic approach to overcome TRAIL-resistant cancers.

## Figures and Tables

**Table 1:** Statistical analysis of MTT cell viability assay results (mean ± SD (%)).

Cell Line							PAM/TRAIL			
	Vehicle	TRAIL	zVAD	DR5/Fc	NAC	PAM	Vehicle	zVAD	DR5/Fc	NAC
HeLa	100 ± 2.3	99.1 ± 1.6	96.4 ± 4.3	92.7 ± 0.5	93.6 ± 3.3	69.0 ± 1.6 **	25.6 ± 0.5 ***	53.3 ± 1.8 ***	55.3 ± 3.9 *	40.2 ± 3.3 ***
A549	100 ± 1.3	99.0 ± 0.6	103.8 ± 6.1	101.0 ± 4.8	106.1 ± 2.7	78.2 ± 0.8 *	39.9 ± 0.2 ***	58.8 ± 1.0 **	61.7 ± 3.2 ***	56.6 ± 2.2 ***
HepG2	100 ± 2.7	96.7 ± 2.8	108.7 ± 5.0	95.8 ± 2.9	108.7 ± 7.6	76.5 ± 3.3 *	32.8 ± 0.6 ***	44.1 ± 0.2 **	45.9 ± 2.3 ***	48.9 ± 2.3 ***

HeLa, A549 or HepG2 cells were pretreated with 25 µM of zVAD, 20 ng/mL DR/Fc or 5 mM NAC for 1 h before TRAIL treatment in the absence or presence of PAM. After 24 h, growth inhibition was monitored via the MTT assay. DMSO was used as a vehicle. Statistical analysis was performed using a one-way ANOVA test followed by Dunnett’s test for comparisons. *, **, and *** indicate significant differences from the control group (* *p* < 0.05, ** *p* < 0.01, *** *p* < 0.001).

**Table 2:** Statistical analysis of apoptosis assay results (mean ± SD (%)).

Cell Line							PAM/TRAIL			
	Vehicle	TRAIL	zVAD	DR5/Fc	NAC	PAM	Vehicle	zVAD	DR5/Fc	NAC
HeLa	7.0 ± 0.5	7.7 ± 0.3	8.2 ± 1.3	8.3 ± 1.3	8.2 ± 0.9	21.1 ± 1.4 *	64.3 ± 1.6 ***	27.4 ± 1.2 *	19.2 ± 1.1	27.2 ± 2.0 *
A549	9.6 ± 0.2	14.9 ± 0.5	11.6 ± 0.6	12.0 ± 0.4	11.6 ± 1.1	24.6 ± 0.4 *	61.3 ± 2.1 ***	31.7 ± 1.4 *	15.6 ± 0.6	29.4 ± 1.4 *

HeLa and A549 cells were pretreated with 25 µM of zVAD, 20 ng/mL DR/Fc or 5 mM NAC for 1 h before TRAIL treatment in the absence or presence of PAM. After 24 h incubation, cell death was determined by FACS analysis following Annexin V and propidium iodide staining. DMSO was used as a vehicle. Statistical analysis was performed using a one-way ANOVA test followed by Dunnett’s test for comparisons. * and *** indicate significant differences from the control group (* *p* < 0.05, *** *p* < 0.001).

## Supplementary Materials

Supplementary materials can be found at https://www.mdpi.com/1422-0067/21/15/5302/s1.

## Abbreviations

TRAIL	Tumor necrosis factor-related apoptosis-inducing ligand
PAM	plasma-activated medium
ROS	reactive oxygen species
RNS	reactive nitrogen species
NAC	N-acetyl-cysteine
GSH	glutathione
DR	death receptor
TNF	tumor necrosis factor
HDF	human dermal fibroblast
c-FLIP	cellular FLICE (FADD-like IL-1β-converting enzyme)-inhibitory protein
CHOP	CCAAT/enhancer binding protein (C/EBP) homologous protein
SOD	superoxide dismutase
PTEN	phosphatase and Tensin homolog deleted on Chromosome 10
mROS	mitochondrial reactive oxygen species
