# Supplementary material for: Non-Thermal Plasma Couples Oxidative Stress to TRAIL Sensitization through DR5 Upregulation
Source: Int J Mol Sci. 2020 Jul 26;21(15):5302. doi: 10.3390/ijms21155302 (PMC7432737; doi:10.3390/ijms21155302)
Supplement: Supplementary file 1 [file ijms-21-05302-s001.zip › ijms-876691-supplementary Proofreading.docx]

**Supplementary figure legends**


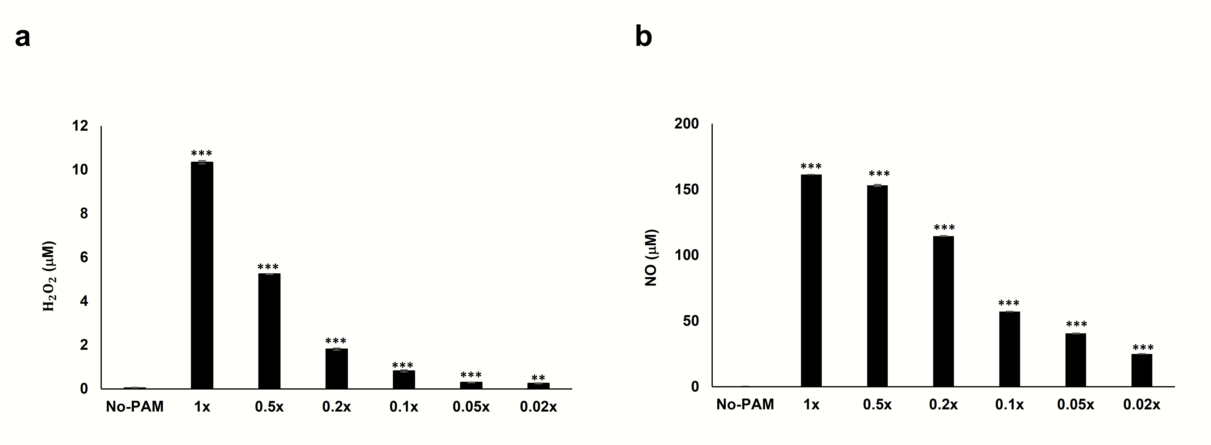


**Supplementary Figure S1.** ROS and RNS in PAM. (**a**) Concentration of H_2_O_2_ in PAM was analyzed by using the Amplex UltraRed hydrogen peroxide assay kit. (**b**) The levels of RNS in PAM were evaluated by the Griess assay. Colorimetric intensity was measured at 540/595 nm. . ** *p* < 0.01, *** *p* < 0.001.


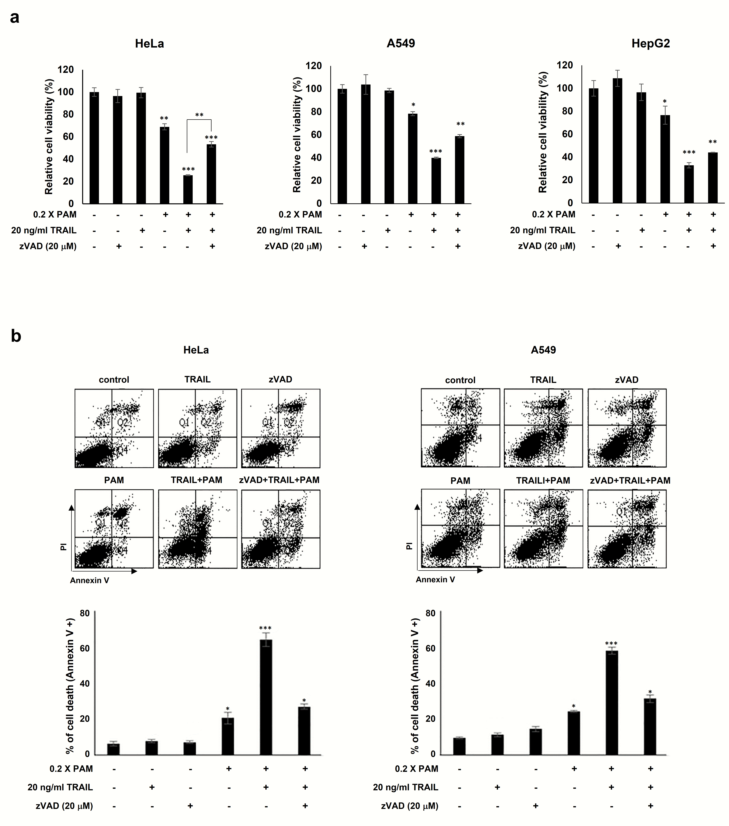


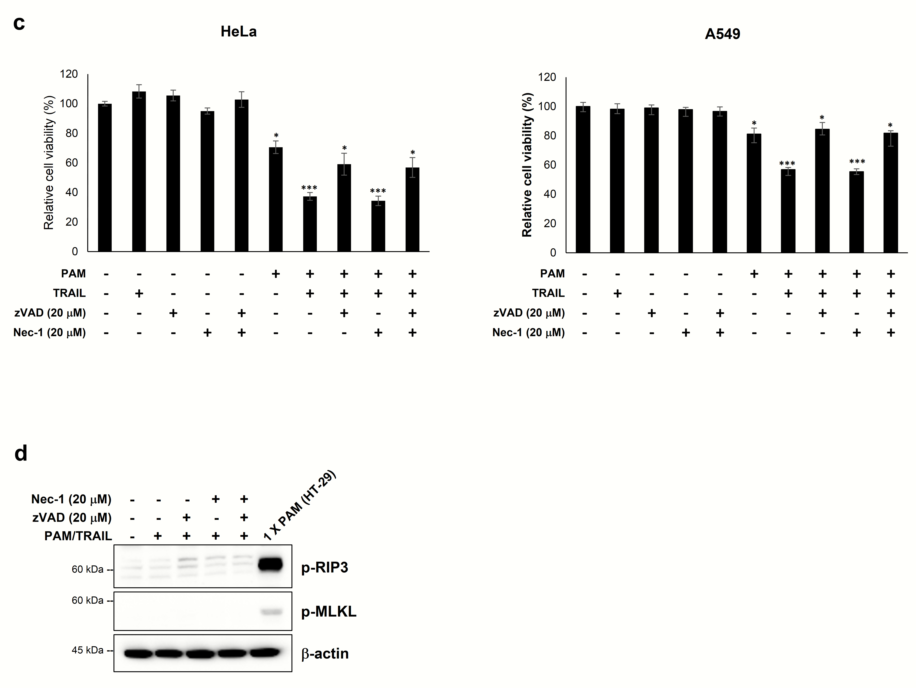


**Supplementary Figure S2.** PAM sensitizes TRAIL-resistant cancer cells to TRAIL-mediated apoptosis. (**a**) HeLa, A549 or HepG2 cells were pretreated with 20 µM of the pan-caspase inhibitor z-VAD-fmk (zVAD) for 1 h before TRAIL treatment in the absence or presence of PAM. After 24 h, growth inhibition was monitored via the MTT assay. (**b**) HeLa and A549 cells were treated as described in (a). Cell death was determined by FACS analysis following Annexin V and propidium iodide staining. Upper panel shows a representative image of FACS analysis. Bottom panel presents statistical analysis of cell death. (**c**) HeLa and A549 cells were pretreated with 20 μM of zVAD and necrostatin-1 (Nec-1) for 1 h before TRAIL treatment in the absence or presence of PAM. After 24 h, growth inhibition observed with MTT assay. (**d**) HeLa cells were pretreated with zVAD and/or Nec-1 for 1 h before TRAIL treatment in the presence of PAM. After 24 h, phosphorylation of RIP3 and MLKL was observed by western blot analysis. 1 X PAM treated HT-29 cell lysate was used as positive control for necroptosis. * *p* < 0.05, ** *p* < 0.01, *** *p* <0.001.


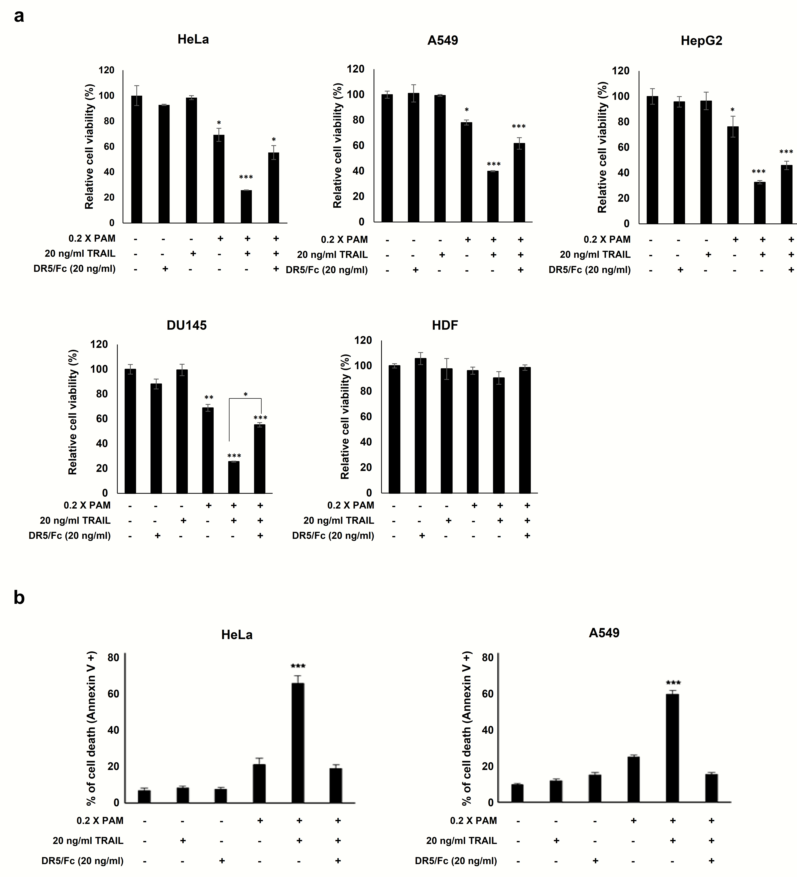


**Supplementary Figure S3.** Combinational treatment with PAM and TRAIL sensitizes TRAIL-resistant cancer cells via DR5 expression. (**a**) DR5-specific blocking chimeric antibody (DR5/Fc) ameliorates PAM/TRAIL-induced growth. HeLa, A549, HepG2, prostate cancer DU145 and human dermal fibroblast HDF cells were pretreated with DR/Fc (20 ng/mL) for 1 h before co-treatment with 0.2 × PAM and TRAIL (20 ng/mL). Growth inhibition after 24 h of treatment by performing the MTT assay. (**b**) HeLa and A549 cells were treated as described in (**a**). Cell death was determined by FACS analysis following Annexin V and propidium iodide staining. Upper panel shows a representative image of FACS analysis. Bottom panel presents statistical analysis of cell death. * *p* < 0.05, ** *p* < 0.01, *** *p* <0.001.


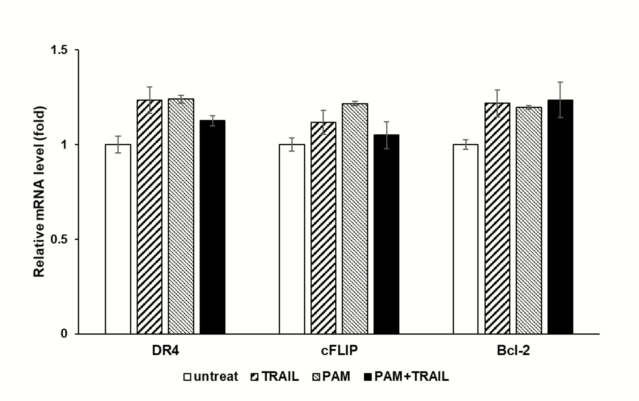


**Supplementary Figure S4.** Treatment with PAM and TRAIL does not affect transcription of DR4, cFLIP and Bcl-2. HeLa cells were treated with 0.2 × PAM, TRAIL (20 ng/mL), or PAM/TRAIL (0.2 × PAM with 20 ng/mL TRAIL) for 24 h. The mRNA levels for indicated genes were determined by qRT-PCR. GAPDH used as an internal control.


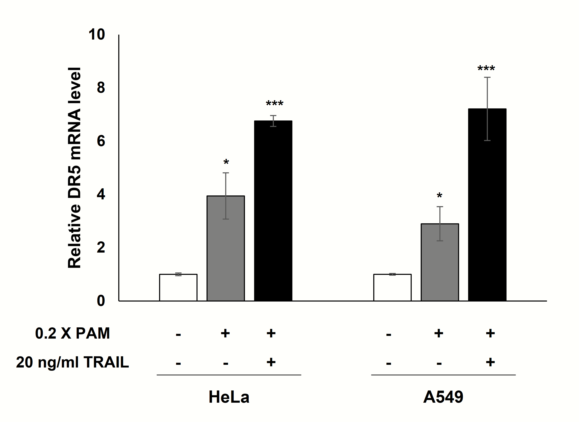


**Supplementary Figure S5.** Co-treatment of PAM with TRAIL upregulates DR5 mRNA in A549 cells. HeLa and A549 cells were treated with 0.2 × PAM or PAM/TRAIL (0.2 × PAM with 20 ng/mL TRAIL). DR5 mRNA levels were determined by qRT-PCR. GAPDH used as an internal control. * *p* < 0.05, *** *p* < 0.001.


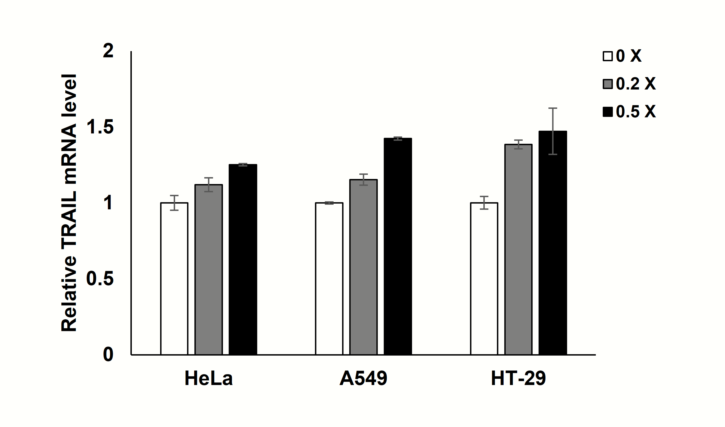


**Supplementary Figure S6.** Treatment with PAM has little effects on the TRAIL mRNA levels. HeLa, A549 and HT-29 cells were treated with 0 ×, 0.2 × and 0.5 × PAM for 16 h. TRAIL mRNA levels were determined by qRT-PCR.


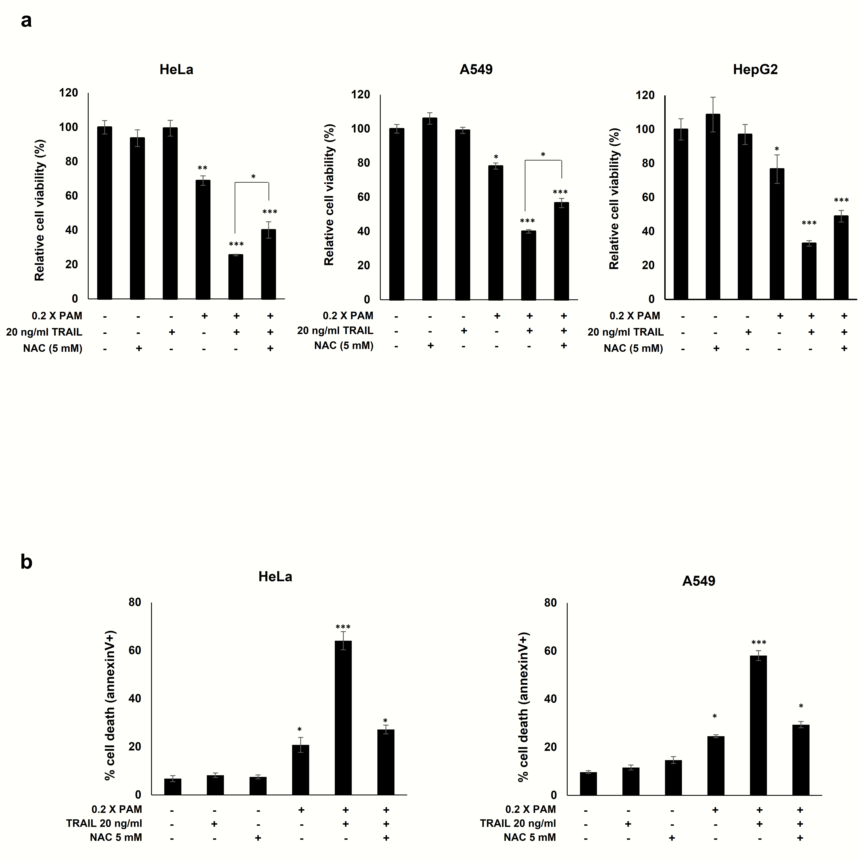


**Supplementary Figure S7.** ROS generation is involved in PAM-mediated TRAIL sensitization. (**a**) HeLa, A549 or HepG2 cells were pretreated with NAC (5 mM) for 1 h before treatment with PAM, TRAIL, or PAM/TRAIL. In further 24 h, cell viability was analyzed by MTT assay. (**b**) HeLa and A549 cells were treated as described in (a), followed by apoptosis assay. * *p* < 0.05, ** *p* < 0.01, *** *p* <0.001.


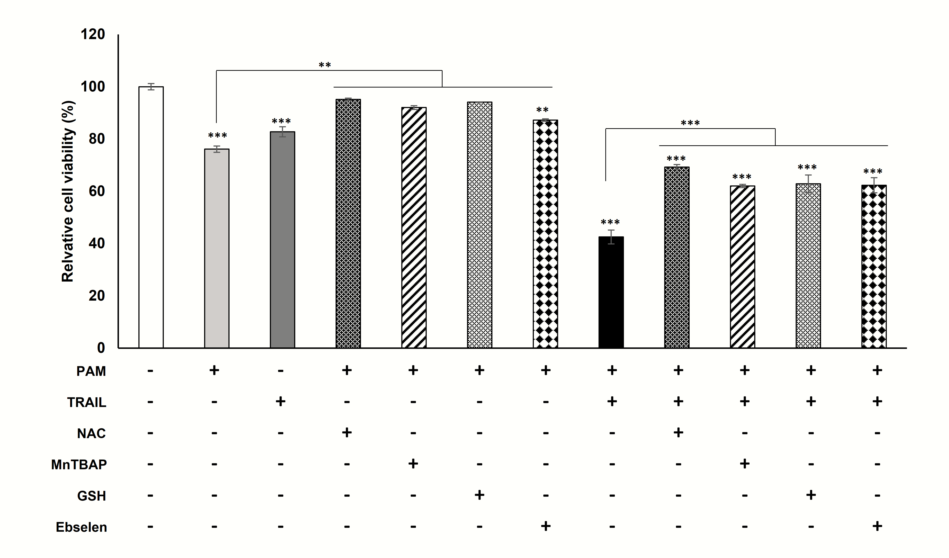


**Supplementary Figure S8**. Antioxidant protects cancer cells from co-treatment-induced apoptosis by blocking ROS accumulation. HeLa cells were pretreated 5 mM N-acetylcysteine (NAC), 5 μM MnTBAP, 5 mM GSH or 10 μM Ebselen for 1 h before treatment with 20 ng/mL TRAIL in the absence or presence of PAM. Growth inhibition was monitored after 24 h, based on the MTT assay. ** *p* < 0.01, *** *p* <0.001.


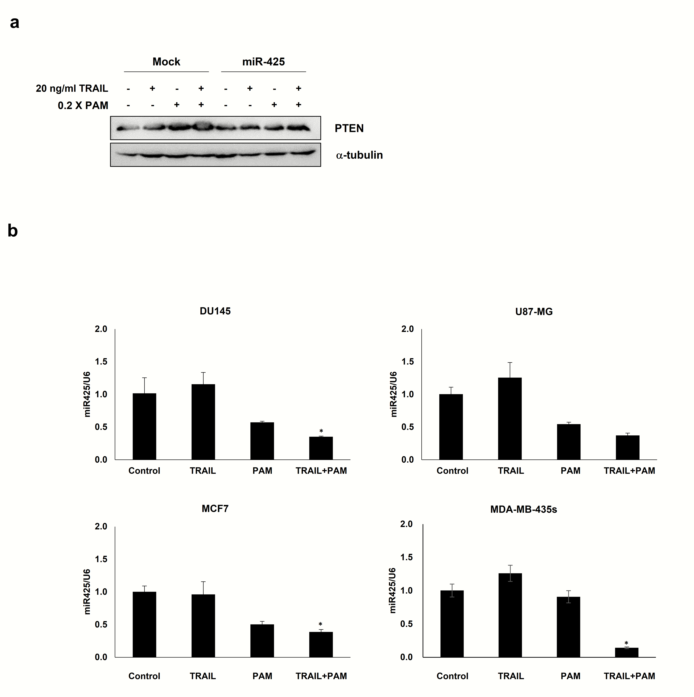


**Supplementary Figure S9.** Combined treatment with PAM and TRAIL induces miR-425 downregulation in different TRAIL-resistant cancer cells. (**a**) miR-425 transfected HeLa cells were treated with 0 or 20 ng/mL TRAIL in a presence or absence of PAM for 24 h. PTEN expression was determined via immunoblot analysis. (**b**) Du145, U87, MCF7, and MDA-MB-435s cells were treated with TRAIL with or without PAM for 24 h. miR-425 expression was analyzed by qRT-PCR. The levels of miR-425 were normalized with U6. * *p* < 0.05.
