# Supplementary figures and images for: Non-Thermal Plasma Couples Oxidative Stress to TRAIL Sensitization through DR5 Upregulation
Source: Int J Mol Sci. 2020 Jul 26;21(15):5302. doi: 10.3390/ijms21155302 (PMC7432737; doi:10.3390/ijms21155302)

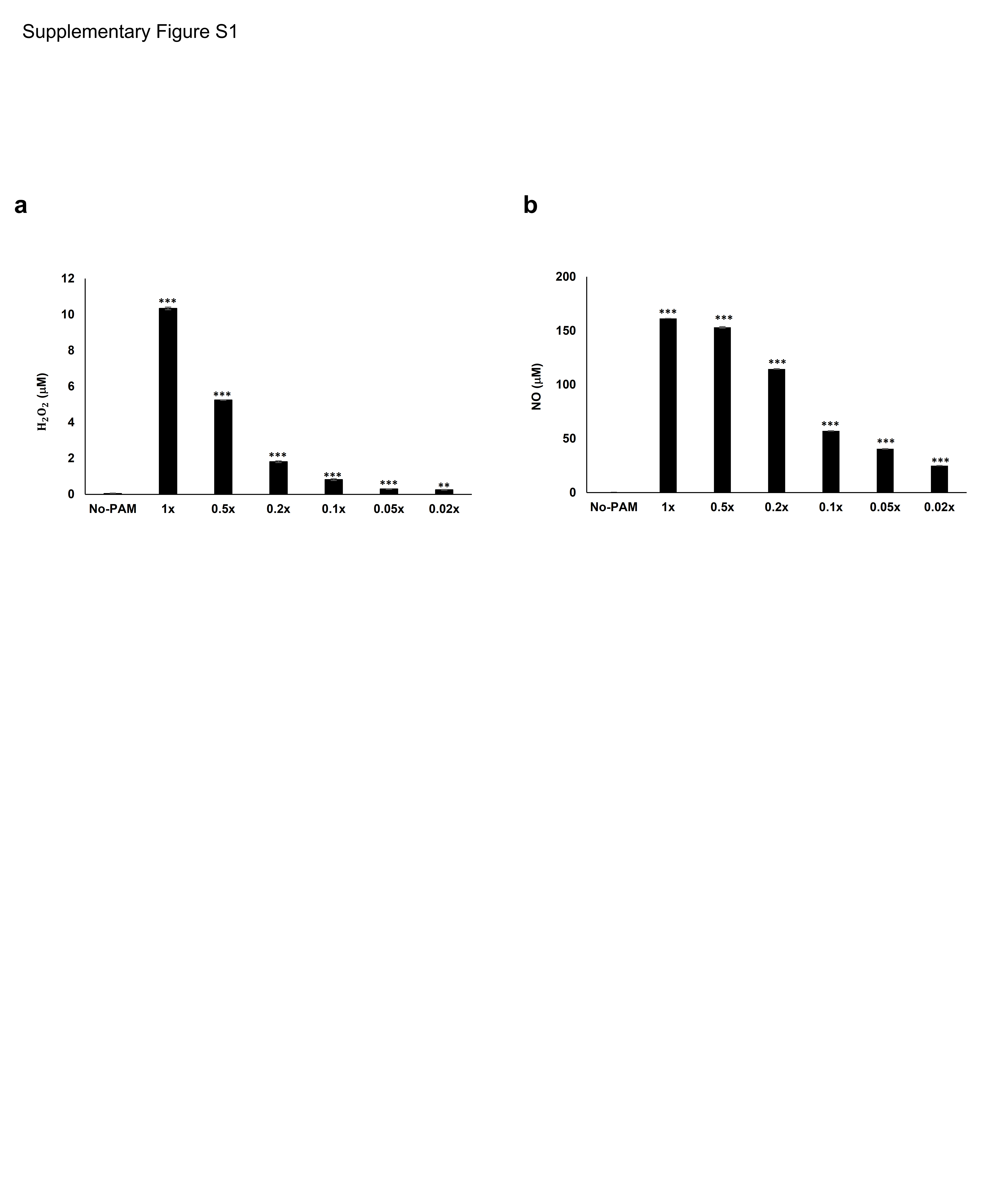

Supplement: Supplementary file 1 [file ijms-21-05302-s001.zip › ijms-876691-supplementary/Supple Figure (IJMS)/Supple FigS1.TIF]

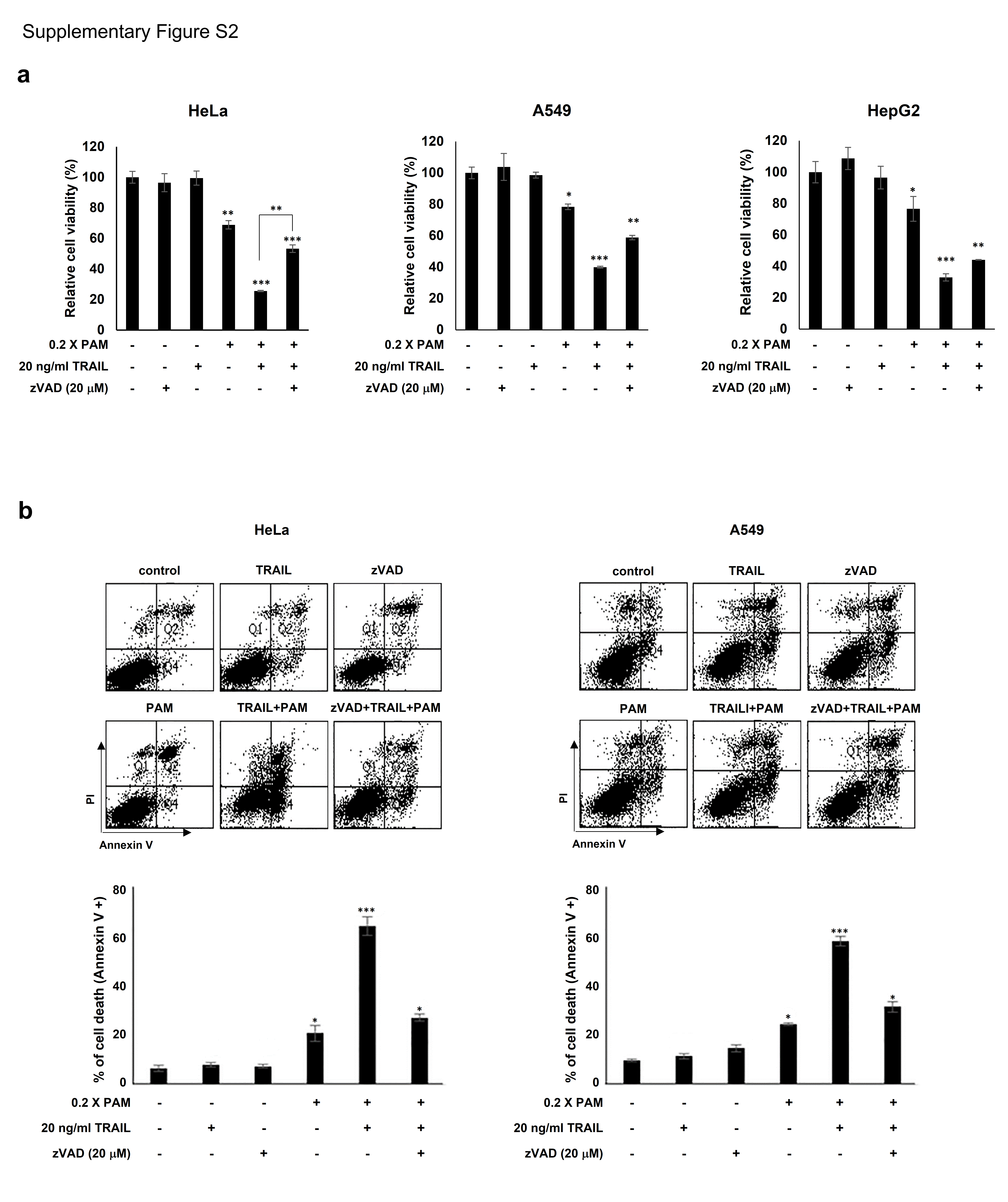

Supplement: Supplementary file 1 [file ijms-21-05302-s001.zip › ijms-876691-supplementary/Supple Figure (IJMS)/Supple FigS2-1.TIF]

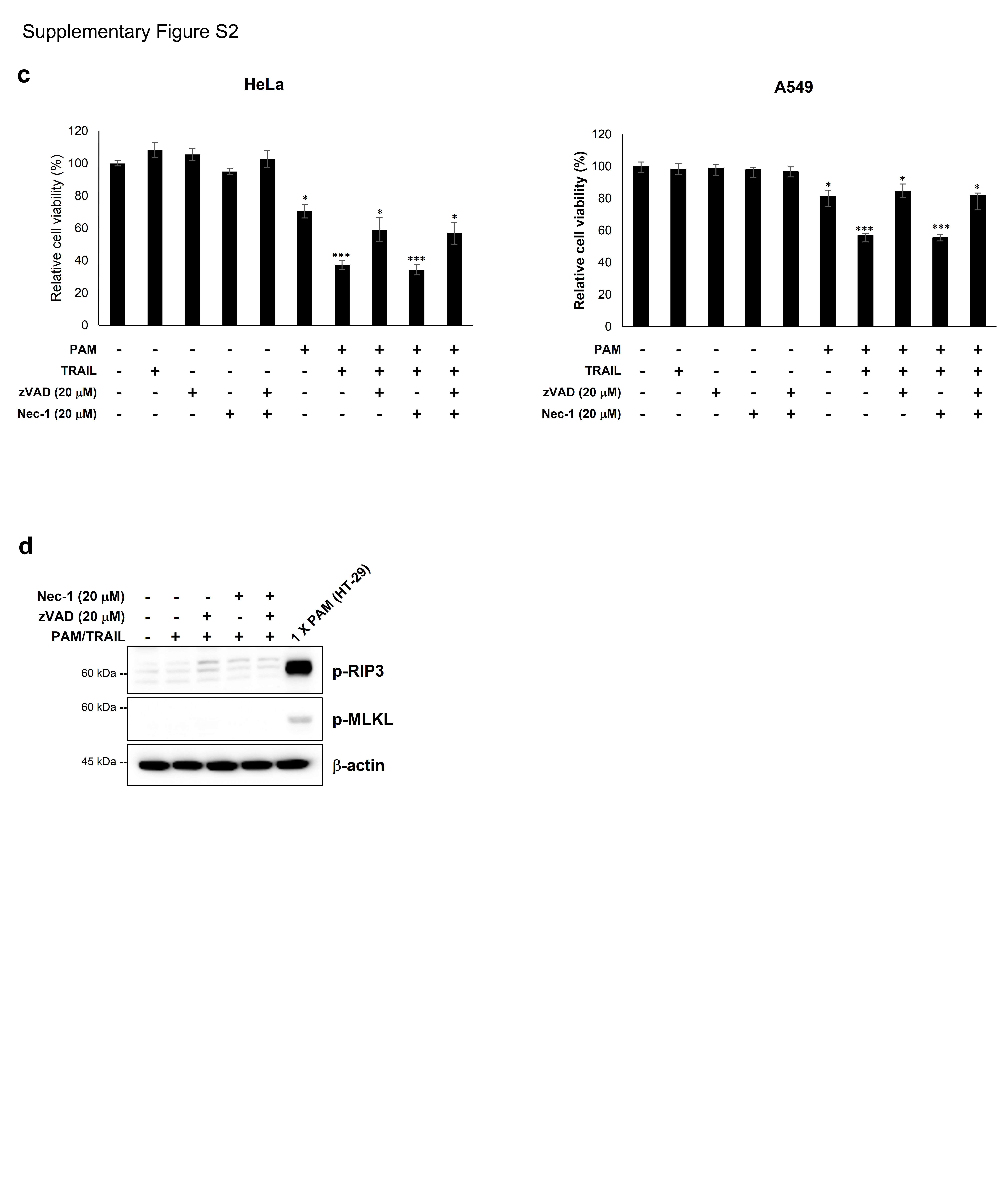

Supplement: Supplementary file 1 [file ijms-21-05302-s001.zip › ijms-876691-supplementary/Supple Figure (IJMS)/Supple FigS2-2.TIF]

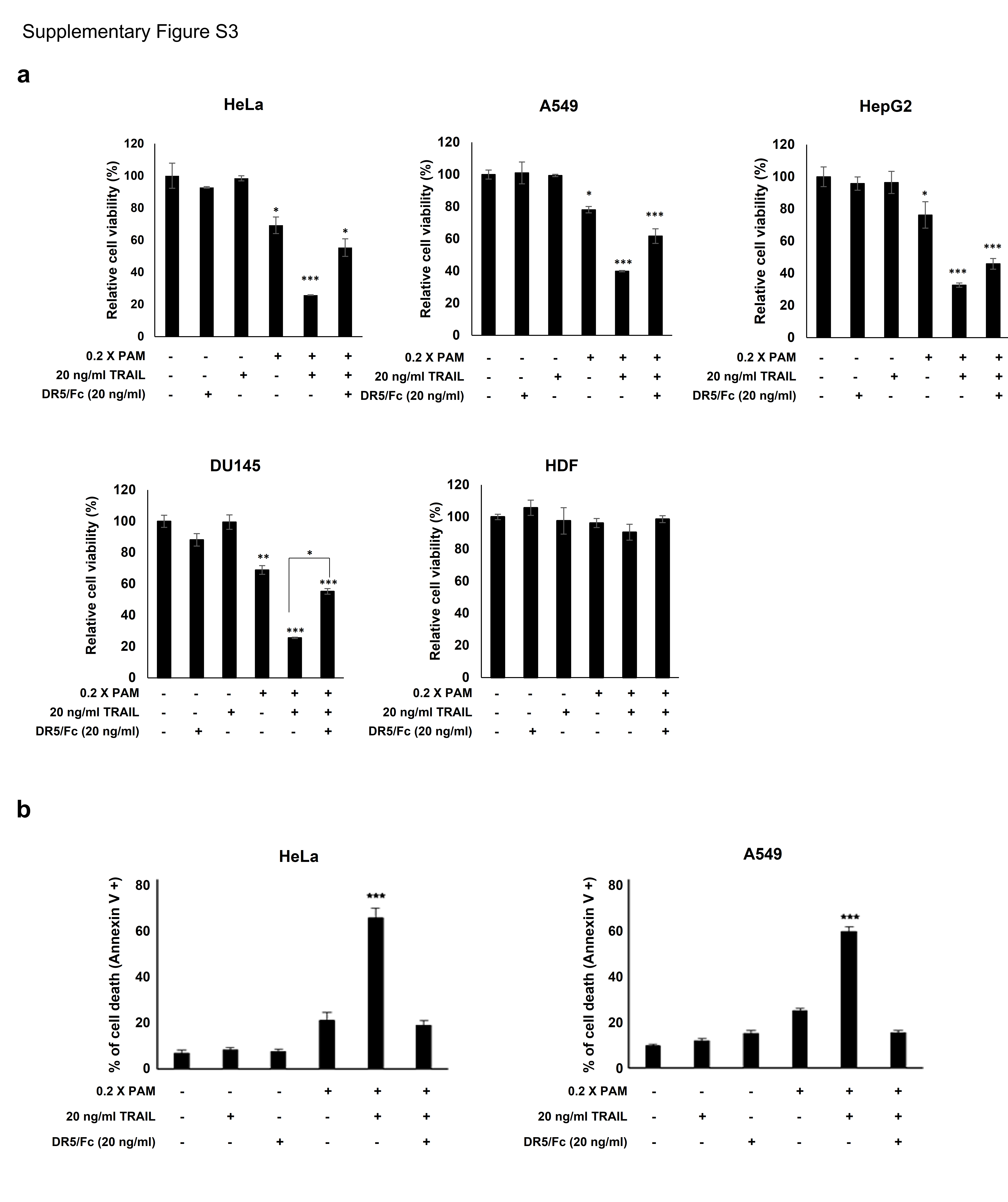

Supplement: Supplementary file 1 [file ijms-21-05302-s001.zip › ijms-876691-supplementary/Supple Figure (IJMS)/Supple FigS3.TIF]

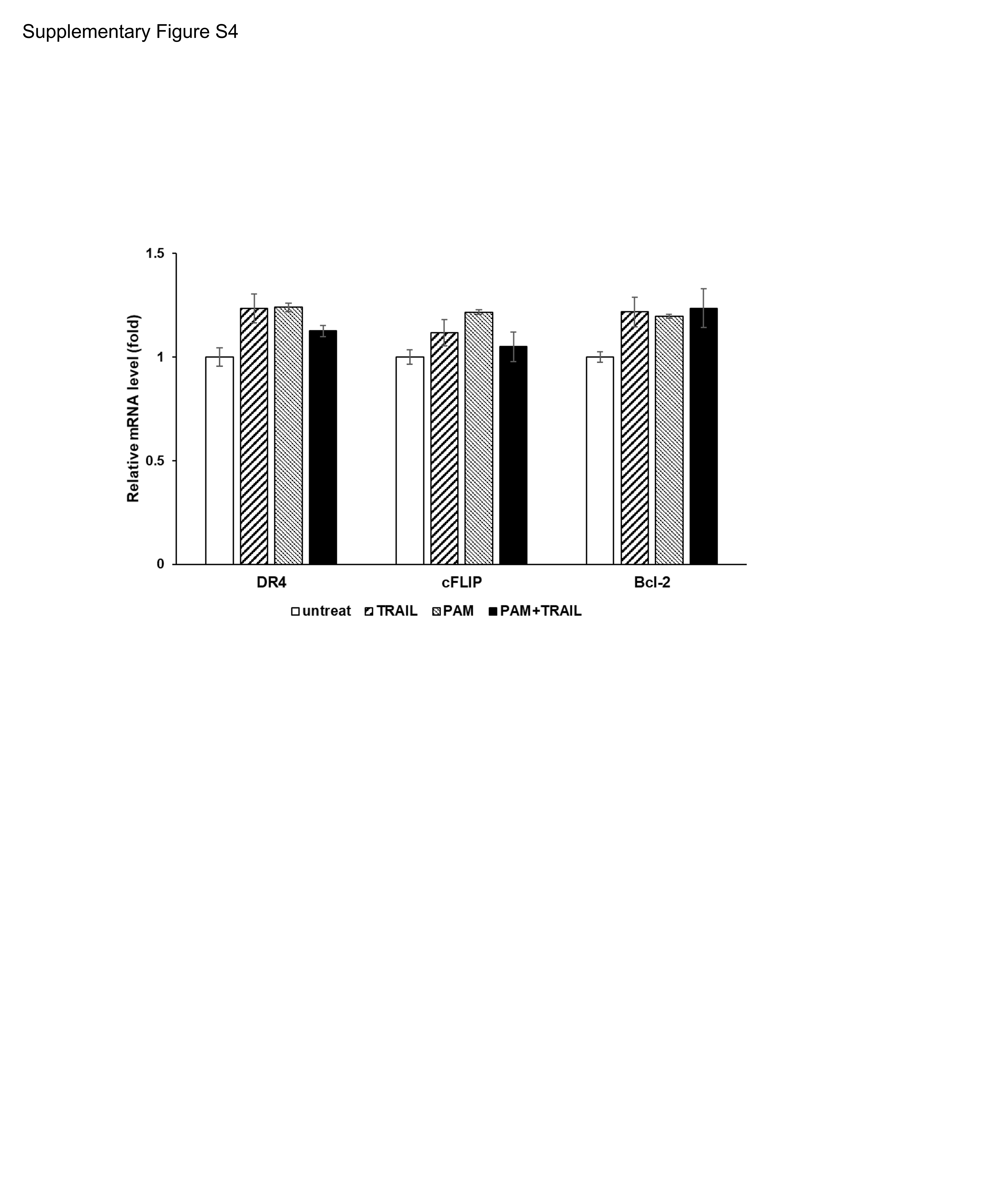

Supplement: Supplementary file 1 [file ijms-21-05302-s001.zip › ijms-876691-supplementary/Supple Figure (IJMS)/Supple FigS4.TIF]

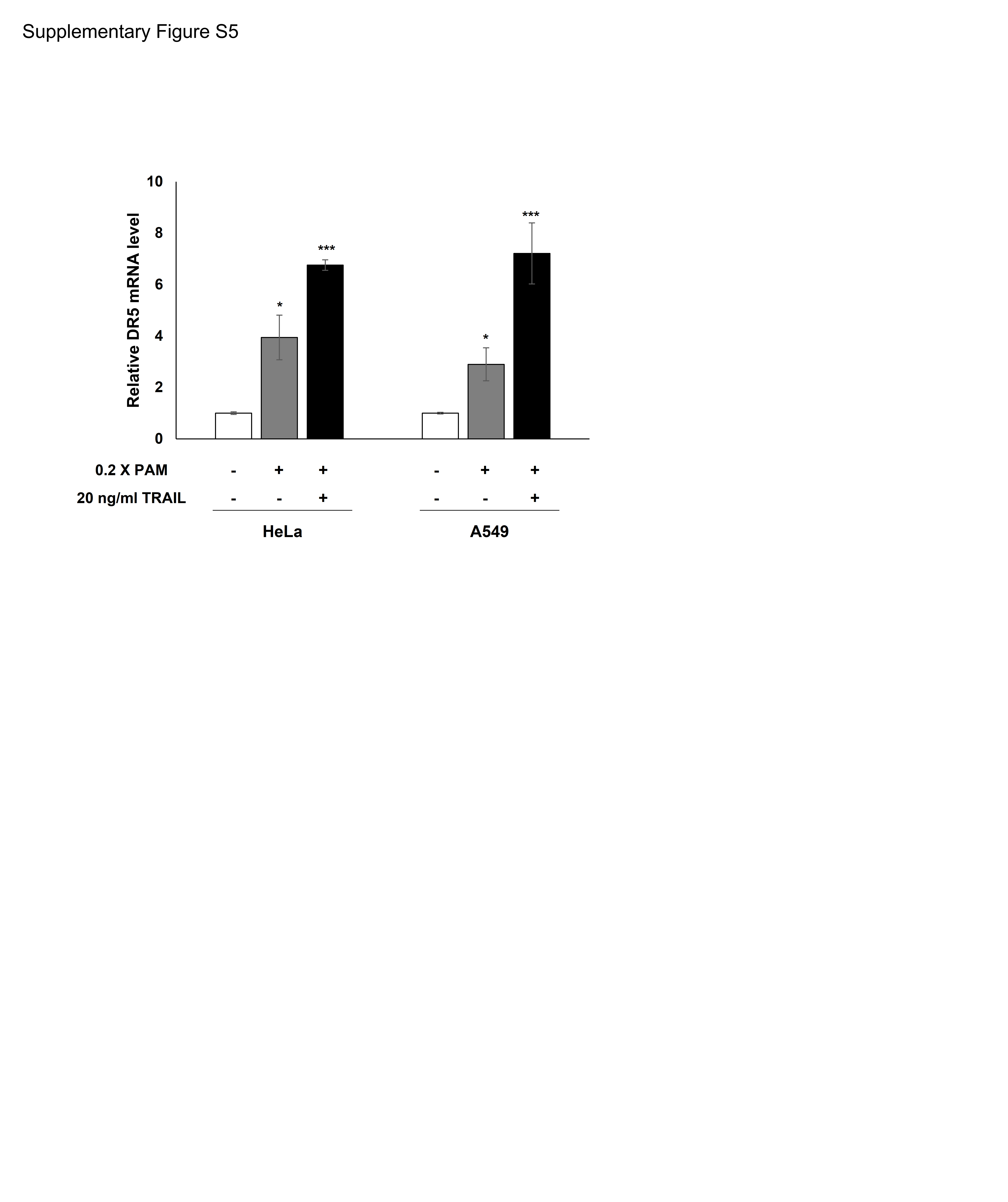

Supplement: Supplementary file 1 [file ijms-21-05302-s001.zip › ijms-876691-supplementary/Supple Figure (IJMS)/Supple FigS5.TIF]

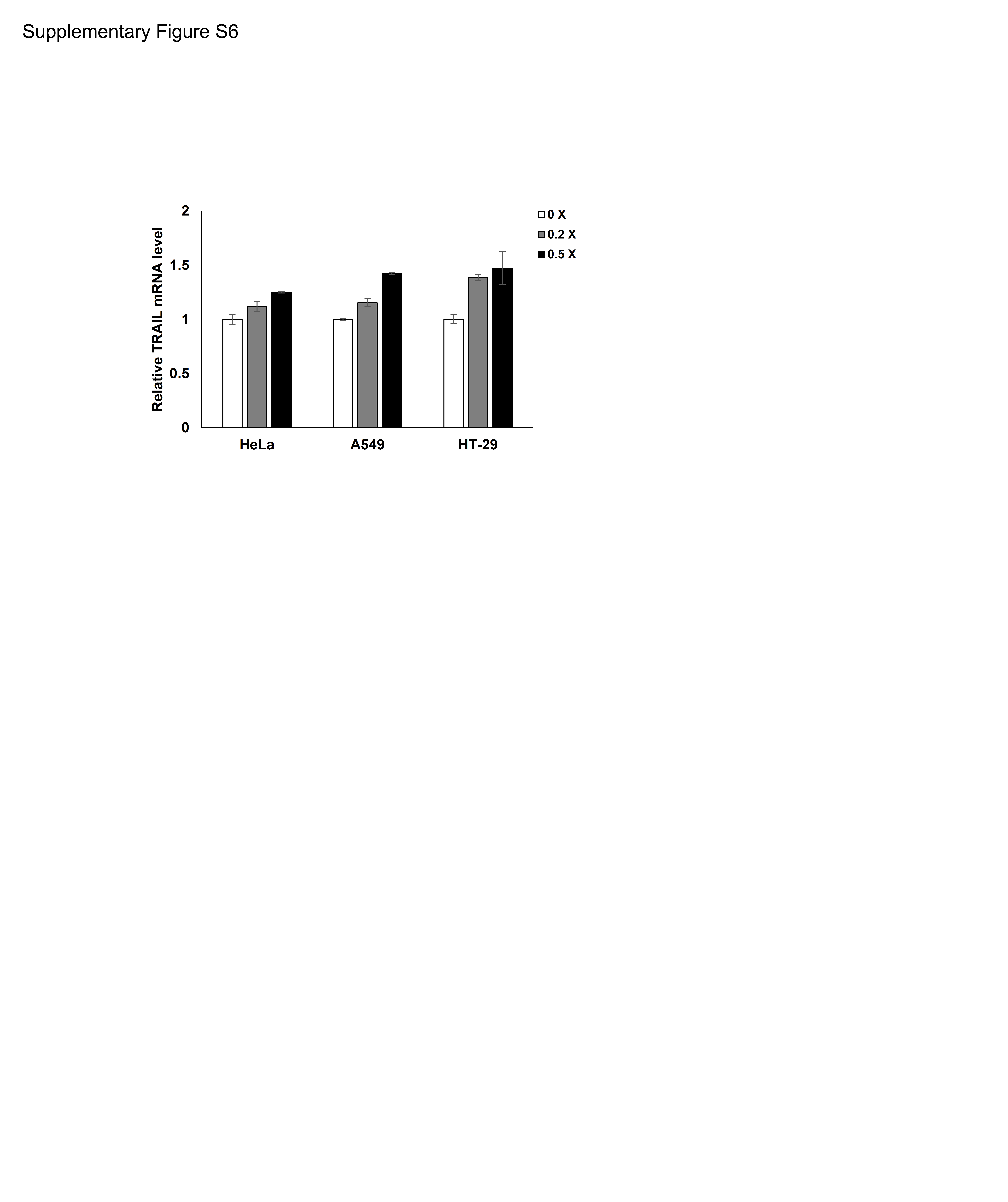

Supplement: Supplementary file 1 [file ijms-21-05302-s001.zip › ijms-876691-supplementary/Supple Figure (IJMS)/Supple FigS6.TIF]

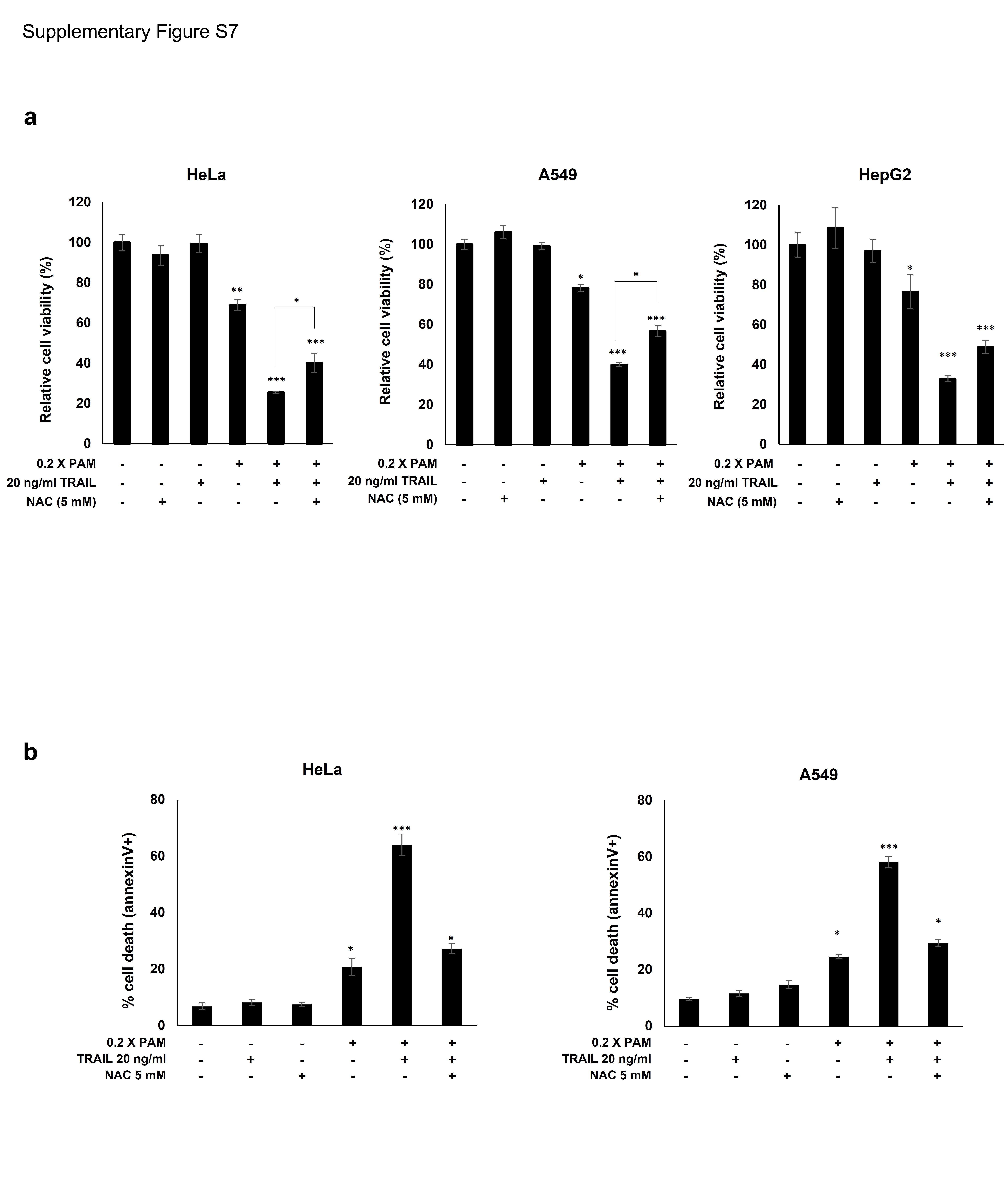

Supplement: Supplementary file 1 [file ijms-21-05302-s001.zip › ijms-876691-supplementary/Supple Figure (IJMS)/Supple FigS7.TIF]

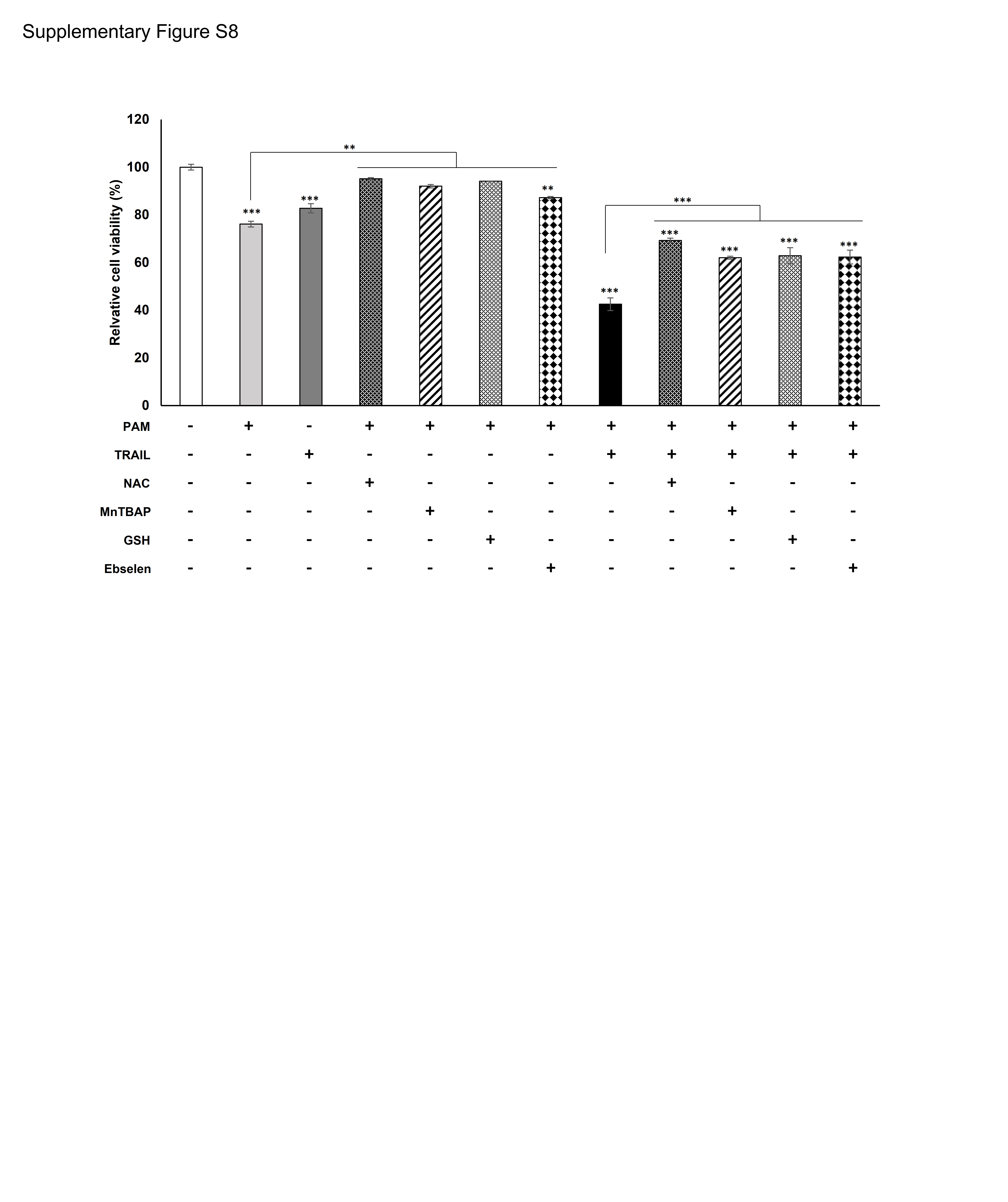

Supplement: Supplementary file 1 [file ijms-21-05302-s001.zip › ijms-876691-supplementary/Supple Figure (IJMS)/Supple FigS8.TIF]

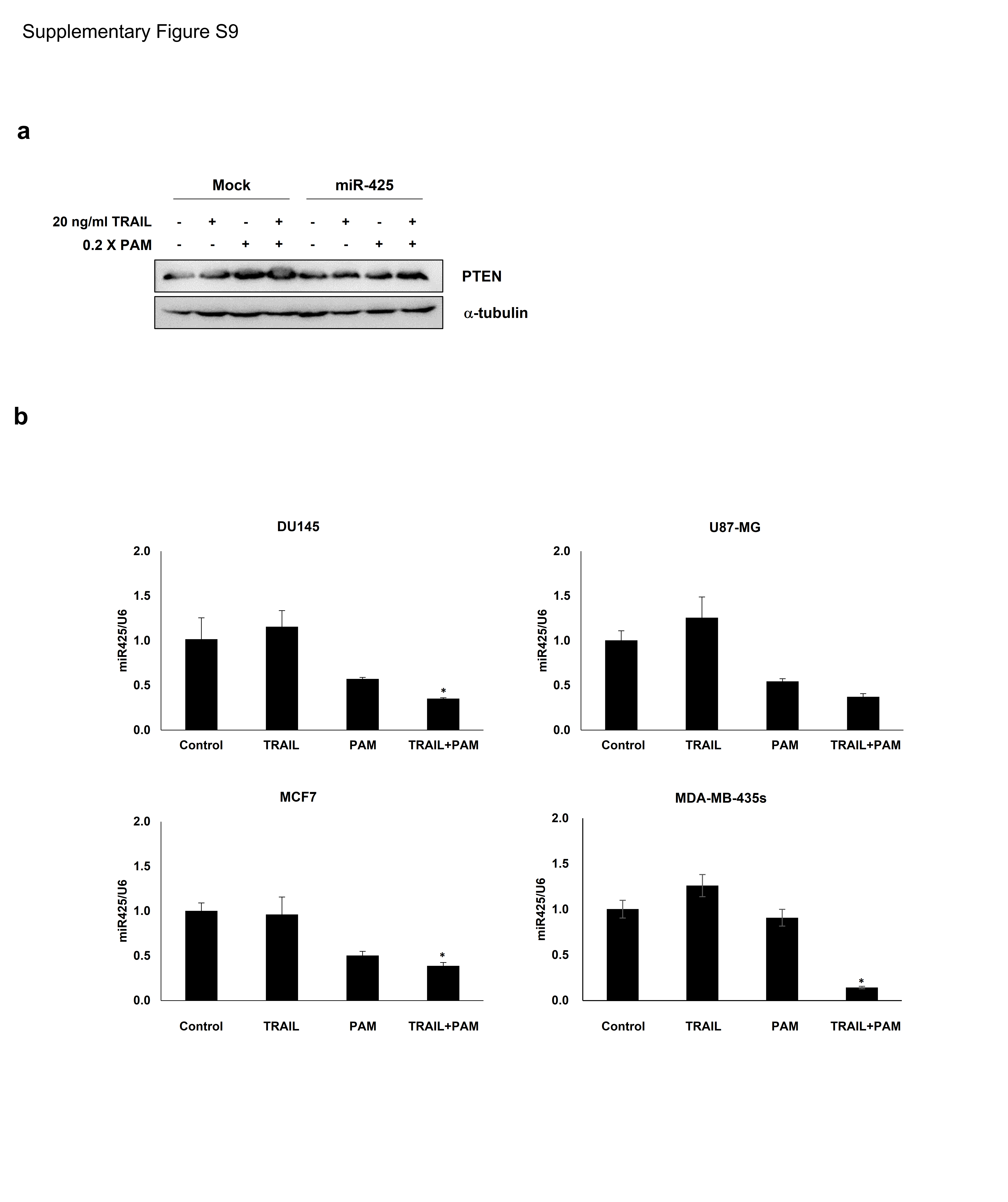

Supplement: Supplementary file 1 [file ijms-21-05302-s001.zip › ijms-876691-supplementary/Supple Figure (IJMS)/Supple FigS9.TIF]
